# Anti-Inflammatory, Antibacterial, Anti-Biofilm, and Anti-Quorum Sensing Activities of the Diterpenes Isolated from *Clinopodium bolivianum*

**DOI:** 10.3390/pharmaceutics16081094

**Published:** 2024-08-20

**Authors:** Luis Apaza Ticona, Ana Martínez Noguerón, Javier Sánchez Sánchez-Corral, Natalia Montoto Lozano, Monserrat Ortega Domenech

**Affiliations:** 1 Organic Chemistry Unit, Department of Chemistry in Pharmaceutical Sciences, Faculty of Pharmacy, University Complutense of Madrid, Plza. Ramón y Cajal s/n, 28040 Madrid, Spain; 2 Department of Organic Chemistry, Faculty of Sciences, University Autónoma of Madrid, Cantoblanco, 28049 Madrid, Spain; 3 Dr. Goya Análisis S.L., Alcalá de Henares, 28805 Madrid, Spain

**Keywords:** *Clinopodium bolivianum*, respiratory infections, antibacterial, biofilm, inflammation, diterpenes

## Abstract

This study reports for the first time the isolation of four diterpenoid compounds: 15-Hydroxy-12-oxo-abietic acid (**1**), 12*α*-hydroxyabietic acid (**2**), (−)-Jolkinolide E (**3**), and 15-Hydroxydehydroabietic acid (**4**) from *Clinopodium bolivianum* (*C. bolivianum*). The findings demonstrate that both the dichloromethane/methanol (DCMECB) extract of *C. bolivianum* and the isolated compounds exhibit significant anti-inflammatory (inhibition of NF-κB activation), antibacterial (primarily against Gram-positive bacteria), and anti-biofilm (primarily against Gram-negative bacteria) activities. Among the isolated diterpenes, compounds **3** and **4** showed notable anti-inflammatory effects, with IC_50_ values of 17.98 μM and 23.96 μM for compound **3**, and 10.79 μM and 17.37 μM for compound **4**, in the HBEC3-KT and MRC-5 cell lines. Regarding their antibacterial activity, compounds **3** and **4** were particularly effective, with MIC values of 0.53–1.09 μM and 2.06–4.06 μM, respectively, against the *S. pneumoniae* and *S. aureus* Gram-positive bacteria. Additionally, these compounds demonstrated significant anti-biofilm and anti-quorum sensing activities, especially against Gram-negative bacteria (*H. influenzae* and *L. pneumophila*). We also explain how compound **3** (BIC = 1.50–2.07 μM, Anti-QS = 0.31–0.64 μM) interferes with quorum sensing due to its structural homology with AHLs, while compound **4** (BIC = 4.65–7.15 μM, Anti-QS = 1.21–2.39 μM) destabilises bacterial membranes due to the presence and position of its hydroxyl groups. These results support the traditional use of *C. bolivianum* against respiratory infections caused by both Gram-positive and Gram-negative bacteria. Furthermore, given the increasing antibiotic resistance and biofilm formation by these bacteria, there is a pressing need for the development of new, more active compounds. In this context, compounds **3** and **4** isolated from *C. bolivianum* offer promising potential for the development of a library of new, more potent, and selective drugs.

## 1. Introduction

Bacterial respiratory infections comprise a group of diseases affecting the respiratory tract, caused by various types of pathogenic bacteria. These infections are a leading cause of illness and death worldwide, affecting individuals of all ages and conditions [1]. The severity of bacterial respiratory infections can vary considerably, from mild cases such as sinusitis to serious and potentially life-threatening diseases such as bacterial pneumonia [2]. Additionally, bacterial infections can vary significantly depending on whether they are caused by Gram-negative or Gram-positive bacteria [3]. In this regard, Gram-negative bacteria such as *Haemophilus influenzae* (*H. influenzae*) and *Legionella pneumophila* (*L. pneumophila*) pose significant medical challenges due to their potential severity and transmission mechanisms. *H. influenzae* can cause various respiratory infections such as otitis media and sinusitis, and in more severe cases, pneumonia [4]. Moreover, *L. pneumophila* is the cause of Legionnaires’ disease, a severe form of pneumonia [5].

Regarding bacterial infections caused by Gram-positive bacteria such as *Streptococcus pneumoniae* (*S. pneumoniae*) and *Staphylococcus aureus* (*S. aureus*), these are largely responsible for pneumonia. *S. pneumoniae* is a major cause of community-acquired pneumonia, as well as otitis media, sinusitis, and meningitis [6]. *S. aureus* infections can cause pneumonia, particularly in hospitalised individuals or those with compromised immune systems [7].

Although effective antibiotics exist to treat respiratory infections caused by *H. influenzae*, *L. pneumophila*, *S. pneumoniae*, and *S. aureus*, the growing resistance to these medicines has become a significant challenge in modern medicine. This resistance is largely due to these bacteria’s ability to form biofilms, a protective structure that makes them less susceptible to antibiotic treatments [8].

Biofilms are communities of bacteria that adhere to living or inert surfaces, and are surrounded by a self-produced extracellular matrix. This matrix, primarily composed of polysaccharides, proteins, and nucleic acids, protects bacteria from the external environment and enables them to resist antimicrobial treatments and the host immune response [9]. Furthermore, this matrix is regulated by quorum sensing systems, which facilitate coordination and cooperation among bacterial cells to achieve collective and efficient resistance.

Quorum sensing is a cellular communication mechanism in bacteria that allows them to coordinate collective behaviours based on their population density. This process depends on the production, release, and detection of small signalling molecules called autoinducers. When the concentration of autoinducers reaches a specific threshold, bacteria detect their presence through receptors and activate or repress the expression of the genes that regulate various community behaviours, such as biofilm formation, virulence, nutrient competition, and resistance factor production [10]. There are several types of quorum sensing systems, distinguished by their signalling molecules and detection mechanisms. In Gram-positive bacteria, peptide autoinducers (AIPs) are commonly used, whereas Gram-negative bacteria often use acyl-homoserine lactones (AHLs) as autoinducers [11].

Simultaneously with bacterial infection and biofilm formation, inflammatory processes are triggered in the body. These inflammatory processes are an immune system response to the presence of pathogens, but they can exacerbate tissue damage and complicate infection. Therefore, in addition to treating bacterial infection and dismantling biofilms, addressing associated inflammation is essential [12]. One approach to treating inflammation is inhibiting key pharmacological targets such as the nuclear factor kappa-light-chain-enhancer of activated B cells (NF-κB), a protein that plays a crucial role in regulating the inflammatory response [13]. NF-κB controls the expression of numerous proinflammatory genes, and its persistent activation can lead to chronic inflammation and tissue damage [14].

Although a wide variety of broad-spectrum antibiotics exists, many bacteria have developed resistance to them, meaning they can tolerate specific concentrations of the drug. This necessitates increasing the doses to achieve the desired effect [15]. However, in the case of biofilms, concentrations that are 100 to 1000 times higher are required to inhibit their formation. This leads to additional challenges given that such high doses cannot be used in human treatments due to their toxicity [16]. Faced with the failure of these treatments, there is a growing need to seek new antibacterial compounds in plant extracts, which represents a promising alternative for developing effective drugs against bacteria [17]. 

In this context, medicinal plants are traditionally used throughout the world as remedies for the treatment of various diseases, including respiratory problems [18]. Natural compounds have demonstrated their beneficial advantages in terms of anti-inflammatory and antibacterial activities [19]. They can restore the clinical application of older antibiotics by increasing their potency and, therefore, avoid the development of drug resistance [20]. Based on their chemical structures, they can be classified into several major groups that include alkaloids, sulphur-containing compounds, polyphenols, and terpenoids [21]. 

Species of the *Clinopodium* genus, belonging to the Lamiaceae family, have attracted increasing interest in scientific research due to their anti-inflammatory, antibacterial, and anti-biofilm properties. For example, Noriega et al. [22] reported that the essential oil extract from *C. brownei* exhibited antimicrobial activity against Gram-positive *Listeria grayi* (*L. grayi*) with a minimum inhibitory concentration (MIC) of 3.84 µg/mL, and against Gram-negative *Proteus vulgaris* (*P. vulgaris*) with an MIC of 4.62 µg/mL. Moreover, different concentrations of essential oil from *C. nubigenum* showed inhibition against *S. aureus* (2.5% inhibition), *Streptococcus mutans* (*S. mutans*), *Streptococcus pyogenes* (*S. pyogenes*), and *S. pneumoniae* (0.3% inhibition) [23]. Extracts from this genus have also shown anti-biofilm activity, such as *C. nepeta*, whose ethyl acetate (EtOAc), *n*-butanol (BuOH), and dichloromethane (DCM) extracts exhibited biofilm inhibitory concentration (BIC) values of 0.5, 1, and 1 mg/mL, respectively, against *Chromobacterium violaceum* (*C. violaceum* in the violacein assay) [24]. These extracts were also able to inhibit the swarm motility of *Pseudomonas aeruginosa* (*P. aeruginosa*) at a concentration of 100 µg/mL, achieving inhibition percentages of 14.89% (DCM extract), 35.42% (EtOAc extract), and 8.27% (BuOH extract) [24].

For our study, we selected the *Clinopodium bolivianum* (Benth.) Kuntze (*C. bolivianum*) plant species, also known by its synonyms *Micromeria boliviana* Benth., *Satureja boliviana* (Benth.) Briq., and *Xenopoma bolivianum* (Benth.) Griseb. [25]. This species, belonging to the Lamiaceae family, is primarily found in the mountains of Bolivia, extending also to eastern Peru and northwestern Argentina [26,27].

In the Central Andes, this aromatic shrub is known as “K’oa” in Aymara, “Muña” in Quechua, and “Oregano of the Incas” in Spanish [28,29], and it is used in traditional Andean medicine [30]. The leaves are inhaled after being slightly crushed to treat colds, coughs (antitussive), fevers (febrifuge), and headaches (analgesic) [31]. Additionally, they are used in infusions (15 g/L) to relieve respiratory pains [32,33] and in decoctions (20 g/L) to treat catarrh and colds [34].

The ethnobotanical relevance of this plant in the treatment of respiratory infections has been corroborated by preliminary phytochemical studies, which have identified monoterpenes as the compounds responsible for this activity. For instance, Mamani Ticona reported the in vitro antibacterial activity of the *C. bolivianum* essential oil (20 μL) against *E. coli* [35]. Similarly, Neira Llerena evidenced that ethanolic and dichloromethane extracts of *C. bolivianum* exhibited antibacterial effects against the bacterial strains *S. aureus* and *S. pneumoniae* at a concentration of 30 mg/mL [36]. Additionally, Claros Paz reported the antibacterial activity of dichloromethane, hydroalcoholic, and aqueous extracts against different strains of *Helicobacter pylori* (*H. pylori*) at a concentration of 0.6 μg/μL (10 μL) [37].

Previous phytochemical studies have revealed that *C. bolivianum* contains a high proportion of terpene-type compounds obtained via the mevalonate acid pathway [38,39], such as the monoterpenes *α*-thujene, *α*-pinene, sabinene, 1,8-cineole, *γ*-terpinene, isomenthone, *α*-terpineol, pulegone, indo bornyl acetate, carvacrol, and caryophyllene, among others [40,41]. Similarly, Dambolena et al. [42] reported the presence of sesquiterpenes using gas chromatography.

In this context, the present manuscript addresses the isolation and characterisation of terpenoid-type compounds from *C. bolivianum* with anti-inflammatory, antibacterial, and anti-biofilm properties, based on their ability to inhibit NF-κB, biofilm formation, and quorum sensing.

## 2. Materials and Methods

### 2.1. General Experimental Procedures

High-purity organic solvents, sourced from Merck, were used in the extraction and fractionation processes, as well as for the isolation of the compounds. Silica gel (SiO_2_ 60 GF254, Merck, Cat. No. 112926-00-8, St. Louis, MO, USA) was used to carry out thin-layer chromatography (TLC). The chromatographic samples were evaluated through two separate methods: a chemical approach using phosphomolybdic acid solution (12Mo_12_O_3_ • H_3_PO_4_, purity ≥ 99.99%, Merck, Cat. No. 51429-74-4, St. Louis, MO, USA) and a physical approach involving UV visualisation with a Spectroline^®^ E-Series lamp, operating at a long wavelength (254 nm), 230 V, NY, USA. A chromatography column was assembled with SiO_2_ (40–63 µm, Merck, Cat. No. 112926-00-8, St. Louis, MO, USA), using the eluents as described in Section 2.2.

NMR measurements were performed using a Bruker Avance DRX 300 spectrometer, with resonance frequencies of 300 MHz for ¹H and 75 MHz for ^13^C. Deuterated chloroform (CDCl_3_, 99.8% atom D, Merck, Cat. No. 865-49-6, DA, DE) was utilised as the solvent. The calibration of the spectra was accomplished by referencing the peaks to the residual solvent.

### 2.2. Extraction and Isolation

The plant species *C. bolivianum* was gathered in June 2019 from the San Juan Huancollo community, located in the Ingavi province, La Paz department, Bolivia (16°35′54.7″ S, 68°57′25.2″ W), at an elevation of 3824 m. The species was botanically identified and verified by the National Herbarium of Bolivia (specimen No. 13924).

The aerial parts of the plants were dried in a hot air oven set to 50 °C for 48 h. Once dried, they were ground into a fine powder (1 kg), which was then subjected to a 30-min decoction at boiling temperature using 2 L of distilled water (dH2O). The resulting aqueous extract (AQECB) was transferred into glass containers and frozen at −38 °C, before being lyophilised using a freeze dryer (Christ Alpha 1–2 LD plus, Benningen, Germany) at −50 °C.

The lyophilised sample (283 g) was subsequently subjected to three separate extractions using 500 mL of *n*-hexane (Hex Merck, Cat. No. 110-54-3, St. Louis, MO, USA) at ambient temperature (25 ± 5 °C) for over 72 h. The solvent was then removed under reduced pressure, yielding 37 g of *C. bolivianum n*-hexane extract (HECB). Following this, an additional three extractions were carried out using a 1:1 *v*/*v* mixture of dichloromethane (DCM ≥ 99.5%, Merck, Cat. No. 75-09-2, St. Louis, MO, USA) and methanol (MeOH 99.8%, Merck, Cat. No. 67-56-1, St. Louis, MO, USA) at the same ambient temperature (25 ± 5 °C) for over 72 h. The solvent was again evaporated under a reduced pressure, resulting in 25 g of dichloromethane/methanol extract from *C. bolivianum* (DCMECB).

Similarly, the DCMECB extract (7 g) was subjected to fractionation using bio-guided column chromatography on SiO_2_ (40–63 μm) with dimensions of 2 cm by 50 cm. The separation was achieved through a stepwise gradient of Hex/EtOAc (99.8%, Merck, Cat. No. 141-78-6, St. Louis, MO, USA) (5:1→1:1 *v*/*v*). This process resulted in the collection of seven fractions (F1→F7). Among these fractions, F1 (85.20 mg), F2 (97.80 mg), F4 (86.70 mg), and F6 (96.10 mg) exhibited the most significant activities.

Subsequently, fraction F1 (80 mg) was separated using column chromatography with SiO_2_ (40–63 μm) in a column measuring 2 cm by 50 cm, with a gradient of Hex/EtOAc (8:1→0:1 *v*/*v*). This process yielded eight sub-fractions (F1A→F1H) with biological activities, among which sub-fraction F1C (Compound **1**, 5.53 mg) showed the most promising results.

Fraction F2 (90 mg) was further separated using a silica gel column (40–63 μm) with dimensions of 2 cm by 50 cm and a gradient of Hex/EtOAc (5:1→0:1 *v*/*v*). This resulted in four sub-fractions (F2A→F2D) that exhibited biological activity, with sub-fraction F2C (Compound **2**, 6.30 mg) showing the highest level of activity.

Similarly, fraction F4 (80 mg) was processed using a silica gel column (40–63 μm) of 2 cm by 50 cm with a gradient of Hex/EtOAc (3:1→0:1 *v*/*v*), producing nine sub-fractions (F4A→F4I) with biological activity. Sub-fraction F4F (Compound **3**, 5.12 mg) was identified as the most active.

Lastly, fraction F6 (90 mg) was separated using column chromatography with SiO_2_ (40–63 μm) in a 2 cm by 50 cm column and a gradient of Hex/EtOAc (2:1→0:1 *v*/*v*), resulting in six sub-fractions (F6A→F6F). Subsequent biological assays revealed that sub-fraction F6B (Compound **4**, 8.64 mg) exhibited the best results.

### 2.3. Spectroscopic Data

#### 2.3.1. 15-Hydroxy-12-oxo-abietic Acid (**1**)

Amorphous powder; ^1^H NMR (CDCl_3_, 300 MHz) δ_H_: 6.88 (s, 1H, H-14), 6.16 (brdd, J = 5.4 and 2.7 Hz, 1H, H-7), 2.55 (brd, J = 14.8 Hz, 1H, H-9), 2.47 (dd, J = 14.8 and 5.0 Hz, 1H, H-11α), 2.32 (t, J = 14.8 Hz, 1H, H-11β), 2.22 (m, 1H, H-6α), 2.15 (m, 1H, H-6β), 1.79 (m, 1H, H-3α), 1.72 (m, 2H, H-1α and H-3β), 1.63 (m, 2H, H-2), 1.44 (s, H, H-17), 1.43 (s, 3H, H-16), 1.31 (s, 3H, H-19), 1.15 (m, 1H, H-1β), 0.90 (s, 3H, H-20); ^13^C NMR (CDCl_3_, 75 MHz) δ_C_: 202.2 (C-12), 182.9 (C-18), 142.2 (C-14), 139.8 (C-13), 135.1 (C-7), 133.4 (C-8), 71.6 (C-15), 48.3 (C-9), 45.7 (C-4), 43.2 (C-5), 38.2 (C-11), 37.7 (C-1), 37.0 (C-3), 34.7 (C-10), 29.3 (C-17), 28.9 (C-16), 26.4 (C-6), 17.7 (C-2), 16.7 (C-19), 14.5 (C-20); HRESIMS m/z 333.2014 [M + H]^+^ (calcd. for C_20_H_29_O_4_^+^, 333.2066). Data were compared to the references [43].

#### 2.3.2. 12α-Hydroxy-abietic Acid (**2**)

Amorphous powder; ^1^H NMR (CDCl_3_, 300 MHz) δ_H_: 5.77 (brs, 1H, H-14), 5.47 (dd, J = 5.4 and 2.5 Hz, 1H, H-7), 4.21 (t, J = 3.0 Hz, 1H, H-12), 2.45 (ps, J = 6.9 Hz, 1H, H-15), 2.35 (m, 1H, H-9), 2.18 (m, 1H, H-5), 2.15 (m, 1H, H-6α), 1.95 (m, 1H, H-6β), 1.90 (m, 2H, H-38α and H-11α), 1.70 (m, 1H, H-3β), 1.61 (m, 2H, H-2), 1.55 (m, 1H, H-1α), 1.30 (m, 1H, H-11β), 1.21 (m, 1H, H-1β), 1.19 (s, 3H, H-19), 1.02 (d, J = 6.9 Hz, 3H, H-17), 0.99 (d, J = 6.9 Hz, 3H, H-16), 0.74 (s, 3H, H-20); ^13^C NMR (CDCl_3_, 75 MHz) δ_C_: 184.3 (C-18), 143.9 (C-13), 134.4 (C-8), 125.7 (C-14), 124.3 (C-7), 66.4 (C-12), 46.2 (C-4), 44.9 (C-5), 43.5 (C-9), 37.9 (C-1), 37.1 (C-3), 34.0 (C-10), 32.5 (C-15), 30.5 (C-11), 25.8 (C-6), 22.3 (C-16), 21.7 (C-17), 18.0 (C-2), 16.6 (C-19), 14.4 (C-20); HRESIMS m/z 319.2133 [M + H]^+^ (calcd. for C_20_H_31_O_3_^+^, 319.2273). Data were compared to the references [44].

#### 2.3.3. (−)-Jolkinolide E (**3**)

Amorphous powder; ^1^H NMR (CDCl_3_, 300 MHz,) δ_H_: 6.32 (th, J = 1.9, 0.9 Hz, 1H, H-14), 5.23 (dddq, J = 6.4, 5.4, 1.9, 1.0 Hz, 1H, H-12), 2.79 (tp, J = 6.9, 1.5 Hz, 1H, H-9), 2.42 (dddd, J = 14.1, 8.4, 5.8, 0.9 Hz, 1H, H-7β), 2.34–2.11 (m, 2H, H-7α and H-11β), 2.11–1.94 (m, 1H, H-5), 1.99–1.86 (m, 2H, H-17, and H-11α), 1.81–1.33 (m, 6H, H-6β, H-1β, H-2β, H-2α, H-6α, and H-3β), 1.33–1.16 (m, 2H, H-1α and H-3α), 0.84 (s, 3H, H-18), 0.83 (s, 3H, H-19), 0.79 (s, 3H, H-20); ^13^C NMR (CDCl_3_, 75 MHz) δ_C_: 175.00 (C-16), 156.41 (C-13), 144.61 (C-8), 116.96 (C-15), 114.88 (C-14), 79.11 (C-12), 53.46 (C-5), 49.67 (C-9), 41.71 (C-3), 38.35 (C-1), 36.99 (C-10), 34.40 (C-7), 33.47 (C-4), 28.42 (C-11), 27.61 (C-18), 27.50 (C-19), 21.65 (C-6), 19.01 (C-2), 16.30 (C-20), 8.73 (C-17); HRESIMS m/z [301.2162 M + H]^+^ (calcd. for C_20_H_29_O_2_^+^, 301.2168). Data were compared to the references [45].

#### 2.3.4. 15-Hydroxy-dehydroabietic Acid (**4**)

Amorphous powder; ^1^H NMR (CDCl_3_, 300 MHz) δ_H_: 7.15 (brs, 2H, H-11 and H-12), 7.08 (brs, 1H, H-14), 2.85 (dd, J = 7.7 and 2.6 Hz, 2H, H-7), 2.35 (brd, J = 13.7 Hz, 1H and H-1α), 2.16 (dd, J = 12.4 and 2.2 Hz, 1H, H-5), 1.90 (m, 1H, H-6α), 1.83 (m, 1H, H-3α), 1.76 (m, 2H, H-2), 1.70 (m, 1H, H-3β), 1.58 (m, 1H, H-6β), 1.52 (m, 1H, H-1β), 1.49 (s, 6H, H-16 and H-17), 1.21 (s, 3H, H-19), 1.14 (s, 3H, H-20); ^13^C NMR (CDCl_3_, 75 MHz) δ_C_: 184.5 (C-18), 147.9 (C-9), 146.0 (C-13), 134.8 (C-8), 124.9 (C-14), 124.1 (C-11), 122.0 (C-12), 72.5 (C-15), 47.4 (C-4), 44.6 (C-5), 37.9 (C-1), 36.9 (C-3), 36.7 (C-10), 31.6 (C-16 and C-17), 30.1 (C-7), 25.1 (C-20), 21.7 (C-6), 18.5 (C-29, 16.2 (C-19); HRESIMS m/z [317.2097 M + H]^+^ (calcd. for C_20_H_29_O_3_^+^, 317.2117). Data were compared to the references [46].

### 2.4. Cytotoxicity and Anti-Inflammatory Activity

#### 2.4.1. Cell Culture

This study utilised the HBEC3-KT (human lung and bronchial epithelial cells, CRL-4051), MRC-5 cells (human lung fibroblasts, CCL-171), and THP-1 cells (human peripheral blood monocytes, TIB-202) cell lines. These cell lines were obtained from the American Type Culture Collection (ATCC, Manassas, VA, USA). The THP-1 cell line served as the negative control to assess the cytotoxicity of the samples.

For cell cultivation, the cells were grown in Dulbecco’s Modified Eagle Medium (DMEM, Merck, Cat. No. D5030, St. Louis, MO, USA), supplemented with 2 mM L-glutamine (≥99% Merck, Cat. No. 56-85-9, St. Louis, MO, USA), 10% fetal bovine serum (FBS, Merck, Cat. No. TMS-016, St. Louis, MO, USA), along with 100 units/mL of penicillin and 100 µg/mL of streptomycin (Merck, Cat. No. P4333, St. Louis, MO, USA) in the culture flasks. The cells were incubated under standard conditions (20–21% O_2_) in a humidified environment (5% CO_2_ at 37 °C).

Stock solutions of the samples (extracts and compounds) were prepared at a concentration of 1 mM using dimethyl sulfoxide (DMSO ≥ 99.9%, Merck, Cat. No. 67-68-5, St. Louis, MO, USA) as the solvent. Dilutions from these stock solutions were then made to achieve concentrations between 100 and 0.20 μg/mL or μM in the culture medium, which contained 0.5% DMSO. A control group consisting only of the culture medium with 0.5% DMSO was also included to determine its potential cytotoxicity.

#### 2.4.2. Cytotoxicity Assay 

Cell viability was evaluated using a colorimetric method on 96-well plates, using 2-(4-iodophenyl)-3-(4-nitrophenyl)-5-(2,4-disulfophenyl)-2*H*-tetrazolium monosodium salt (WST-1, Merck, Cat. No. 5015944001, St. Louis, MO, USA) as the reagent [47]. Actinomycin D (ACTD ≥ 95%, Merck, Cat. No. 50-76-0, St. Louis, MO, USA) was used as a positive control, with a CC_50_ value of 0.01 μg/mL for the extracts, or 0.008 μM for the compounds. The HBEC3-KT, MRC-5, and THP-1 cell lines were cultivated in 96-well plates at 37 °C with 5% CO_2_ until they reached over 90% confluency (3 × 10^3^ cells per well). The cells were then exposed to varying concentrations of the samples (ranging from 100 to 0.20 μg/mL or μM) for 24 h at 37 °C with 5% CO_2_, in DMEM containing 8% FBS. Following this incubation period, 10 μL of WST-1 reagent, diluted 1:4 in phosphate buffer, was added to each well, and the plates were incubated for a further 4 h. The cell viability was then assessed by measuring absorbance at 450 nm using an enzyme-linked immunosorbent assay (ELISA) microplate reader (Anthos 2020, Version 2.0.5, Biochrom Ltd., Cambridge, UK).

#### 2.4.3. NF-κB Inhibition Assay

The NF-κB inhibition assay is based on evaluating the ability of an extract or compound to inhibit the activity of NF-κB, and it is a crucial protein in the regulation of the inflammatory response. In this assay, cells that respond to NF-κB activation were cultured and exposed to our extracts and compounds. After inducing NF-κB activation, typically with a pro-inflammatory agent such as lipopolysaccharide (LPS), our extracts or compounds were administered at different concentrations to observe whether they can inhibit this activation.

To assess the anti-inflammatory potential of the samples at the same concentrations used in the cell viability assay, an NF-κB inhibition assay was used following the approach established by Apaza Ticona et al. [47]. Cells were seeded in 96-well plates at a density of 3 × 10^3^ cells per well. Celastrol (CEL ≥ 98%, Merck, Cat. No. 34157-83-0, St. Louis, MO, USA) was utilised as a positive control, with an IC_50_ value of 3.34 μg/mL for the extracts, or 7.41 μM for the compounds. Absorbance was quantified at 450 nm using a microplate reader.

### 2.5. Antibacterial Assays

Before delving into the specific assays, it is crucial to highlight the importance of evaluating the antibacterial activity of extracts or compounds, especially against resistant bacteria and opportunistic pathogens in hospital settings. Assays such as the microdilution method allow us to determine the MIC required to inhibit bacterial growth. In addition to this inhibition, it is also essential to evaluate the ability of the extracts or compounds to interfere with biofilm formation and quorum sensing, which are two key processes for bacterial coordination and virulence. These studies are vital for reducing the capacity of bacteria to form adhered communities (biofilms) on surfaces, which are difficult to eradicate and are associated with persistent infections.

#### 2.5.1. Bacteria

For this study, a variety of bacterial strains were used, including *H. influenzae* (strain 33391), *L. pneumophila* (strain 33152), *C. violaceum* (strain 12472), *S. pneumoniae* (strain BAA-2298), and *S. aureus* (strain BAA-3113). These strains were procured from the American Type Culture Collection (ATCC, Manassas, VA, USA). They were selected due to their significance as major opportunistic pathogens implicated in serious hospital-acquired infections [48,49].

#### 2.5.2. Broth Microdilution Method-MIC

To assess the MICs of the extracts and compounds, a microdilution assay was carried out in 96-well plates (Cellstar^®^, Greiner Bio-One, Frickenhausen, Germany). Initially, a bacterial culture was prepared using a Mueller–Hinton broth (MHB, Merck, Cat. No. 70192, St. Louis, MO, USA), which was inoculated and incubated overnight at 37 °C. From this culture, 4 to 6 colonies were isolated, suspended in sterile 0.85% saline to match a 0.5 McFarland turbidity standard, and then diluted 100-fold with cation-adjusted MHB to reach a concentration of 1 × 10^6^ CFU (Colony-Forming Units) per mL.

Following preparation, 180 μL of this bacterial suspension was added to each well of the microdilution plates. To test the extracts or compounds, 20 μL of each sample, at concentrations identical to those used in the viability assays, was introduced into the wells. The plates were then incubated for 48 h at 37 °C [50]. Ofloxacin (OFX, Merck, Cat. No. 82419-36-1, St. Louis, MO, USA) was included as a positive control, showing a MIC of 10.01 µg/mL for the extracts, or 27.67 μM for the compounds. This testing was conducted on the bacterial strains *H. influenzae*, *L. pneumophila*, *S. pneumoniae*, and *S. aureus*.

#### 2.5.3. Inhibition of Biofilm Formation

To assess the efficacy of the samples in inhibiting biofilm formation (at concentrations identical to those used in the viability assays), the biofilm inhibitory concentration (BIC) was measured using the approach outlined by Perona et al. [51]. For comparison, chlorhexidine digluconate (CHG, Merck, Cat. No. 18472-51-0, St. Louis, MO, USA) was used as a reference control, with a BIC of 2000 μg/mL for the extracts, or 2.23 μM for the compounds. This assay was applied to the bacterial strains *H. influenzae*, *L. pneumophila*, *S. pneumoniae*, and *S. aureus*.

#### 2.5.4. Anti-Quorum Sensing Activity

The potential of the samples to inhibit bacterial communication (at concentrations identical to those used in the viability assays), known as quorum sensing, was assessed by determining the quorum sensing inhibitory concentration (Anti-QS). This evaluation was conducted according to the procedure outlined by Perona et al. [51]. As a positive control, (*Z*)-4-Bromo-5-(bromomethylene)-2(5*H*)-furanone (Furanone C-30, Merck, Cat. No. 247167-54-0, St. Louis, MO, USA) was used, which exhibited an Anti-QS value of 3 μg/mL for the extracts, or 11.81 μM for the compounds. The bacterial strains used in this assay included *H. influenzae*, *L. pneumophila*, *S. pneumoniae*, and *S. aureus*.

#### 2.5.5. Violacein Inhibition Assay

The samples (at concentrations identical to those used in the viability assays) were tested for their ability to inhibit violacein production by determining the violacein inhibitory concentration (CIV), in line with the method outlined by Perona et al. [51]. Ciprofloxacin hydrochloride (CIP, Merck, Cat. No. 86393-32-0, St. Louis, MO, USA) was used as a reference standard, showing a CIV of 1.51 μM for the compounds. The experiment was conducted on the *C. violaceum* strain.

### 2.6. Statistical Analysis

The experiments were repeated three times for accuracy. To assess the impact of different treatments (both concentration and time) on optical density, a two-way ANOVA was conducted with a stringent significance threshold of 0.001. All statistical evaluations were carried out using GraphPad Prism Software version 9.0.0. (86) for Mac, developed by Dennis Radushev (1994–2020, La Jolla, CA, USA, www.graphpad.com).

## 3. Results

### 3.1. Extraction, Isolation, and Characterisation of Compounds

After two extractions from the lyophilised extract of *C. bolivianum* (Figure S1), a spectrophotometric analysis was conducted. Through this analysis, it was observed that the chemical composition of *C. bolivianum* and its extracts is complex. When analysing the results of the NMR spectra, it can be stated that all the signals in the ^1^H NMR spectra correlated with one or more compounds. Each extract had different major compounds, and in the ^1^H NMR spectrum of AQECB, a higher number of signals was observed in the region between 3.0 and4.0 ppm, corresponding to the sugar region. Since the sugars found were rhamnose and glucose (with four or five free hydroxyl groups, respectively), which can bind to phenolic acids and flavonoids, this caused the region to have higher intensity than the aromatic region.

In the case of the ^1^H NMR spectra of HECB, a significant number of aliphatic signals (between 0.8 and 2.0 ppm) was observed, which could correspond to essential oils and predominantly hydrocarbon compounds with limited functional groups (Figure S2). Additionally, the ^13^C NMR spectrum of HECB showed characteristic signals of *sp3* and *sp2* carbons with no apparent binding to heteroatoms (Figure S3). Finally, the ^1^H NMR spectra of DCMECB showed a higher number of aliphatic (0.5–2.5 ppm), allylic (5.0–5.7 ppm), and aromatic (6.8–7.1 ppm) signals, thus demonstrating the presence of more complex chemical structures (compounds from the mevalonate pathway) with biological activity (Figure S4). Based on the results, DCMECB was fractionated, leading to the isolation and characterisation of three compounds.

When analysing the NMR spectra, we can determine that compounds **1** and **2** are abietane-type diterpenes, as they possess two endocyclic double bonds at positions C7-C8 and C13-C14. The locations of these double bonds were established using HMBC spectra (^3^*J*_H,C_), which showed correlations between H-7 and C-5, C-9, and C-14; and between H-14 and C-7, C-9, and C-15. However, the difference between compounds **1** and **2** lies in the substitutions at positions C-12 and C-15.

**Figure 1:**
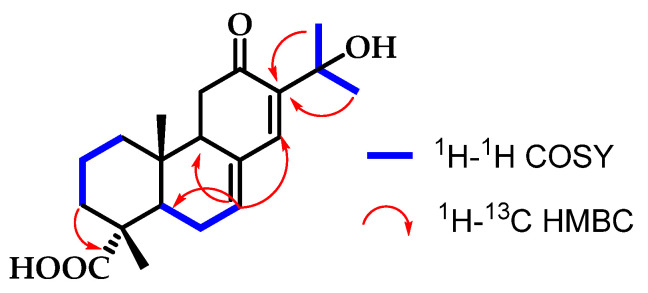
Correlations established in compound **1**.

Compound **1** has a substitution at C-15 (*δ*_C_ 71.6), and at C-12 it has a carbonyl group. This carbonyl group appears to result from the oxidation of the OH group present in compound **2**. The chemical shift of C-12 at 202.2 ppm confirms it is a ketone due to the significant deshielding of the carbon atom C-12. Additionally, the position of the ketone at C-12 was established using the two-dimensional HMBC spectrum, which showed connectivity between C-12 and the protons H-9 and H-14 (Figure 1).

**Figure 2:**
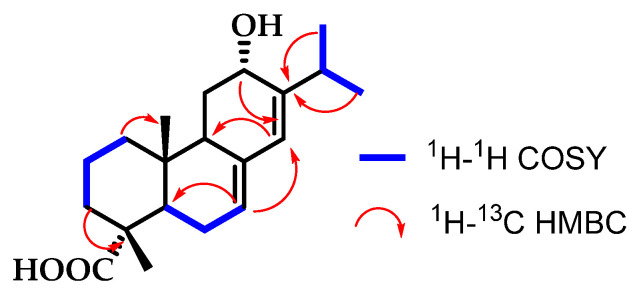
Correlations established in compound **2**.

Compound **2** did not exhibit any substitution at position C-15, as the proton H-15 resonated as a septuplet at *δ*_H_ 2.45 (*sp*, *J* = 6.9 Hz), coupling with the six protons of the methyl groups CH_3_-16 and CH_3_-17. However, a hydroxyl group was observed in the *α* position at C-12, due to the chemical shift of H-12 (*δ*_H_ 4.21), which resonated as a triplet (*J* = 3.0 Hz) given its coupling with the two protons CH_2_-11 of the methylene group. Additionally, H-12 showed correlations in the HMBC spectrum with carbons C-9, C-14, and C-15. The *α* position of the OH was determined by the chemical shift of C-12 (*δ*_C_ 66.4), and by the coupling constant of H-7*β* with the two protons H-6 (Figure 2).

**Figure 3:**
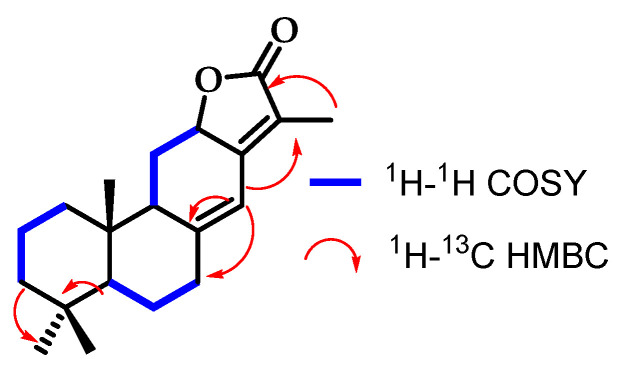
Correlations established in compound **3**.

Compound **3** exhibits three signals corresponding to methyl groups: H-18 (*δ*_H_ 0.84 ppm), H-19 (*δ*_H_ 0.83 ppm), and H-20 (*δ*_H_ 0.79 ppm). Additionally, a decahydronaphthalene bicyclic (1–10) ring system was identified due to its similarity to compound **4**, which shares the same skeleton. However, it was observed that the third ring was not aromatic, as it featured allylic protons such as H-11 (*δ*_H_ 1.86–2.84 ppm), and olefinic protons like H-14 (*δ*_H_ 6.32 ppm). Comparing it with compound **4**, which has an aromatic third ring, distinct chemical shifts were observed in ^1^H NMR and ^13^C NMR for positions 11 and 14: *δ*_H_ 7.15/*δ*_C_ 28.42 ppm and *δ*_H_ 6.32/δC 114.88 ppm, respectively. This suggests that H-14 is olefinic in nature, but H-11 is not conjugated, indicating that it does not form part of an aromatic ring. The presence of a lactone ring was confirmed by the signal at *δ*_C_ 175.00 ppm in the ^13^C NMR spectrum, characteristic of a cyclic ester carbonyl. Additionally, a mass spectrometry analysis was conducted to unequivocally confirm the compound’s presence. Once the structural determination was completed, crystallisation was performed to remove any potential impurities (Figure 3).

**Figure 4:**
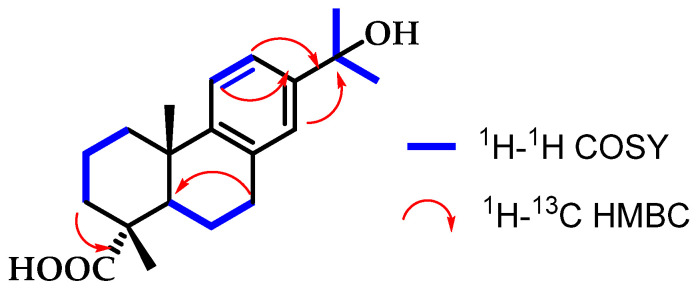
Correlations established in compound **4**.

Compound **4** is a dehydroabietane diterpene. This compound exhibits characteristic signals of a trisubstituted benzene ring in both ^13^C NMR and ^1^H NMR spectra. The chemical shifts of the three aromatic protons, along with their coupling constants, indicate a 1,2,4-trisubstituted benzene ring. Additionally, two methyl groups (H-16/H-17) corresponding to an isopropyl alcohol structure were observed at *δ*_H_ 1.49 ppm. This same pattern was observed in the first compound, where methyl groups 16 and 17 appeared at a chemical shift of *δ*_H_ 1.44–1.43 ppm. Furthermore, a hydroxyl group was located at position C-15, observed as a quaternary carbon at *δ*_C_ 72.5 in the ^13^C NMR spectrum. C-15 also showed correlations in the HMBC spectrum with protons H-12 (*δ*_H_ 7.15) and H-14 *δ*_H_ 7.8). Finally, the presence of the OH group at C-15 affects the chemical shifts of the aromatic protons H-11 and H-12, causing them to resonate at the same chemical shift and thus behave as equivalent protons, although they are not (Figure 4).

**Figure 5:**
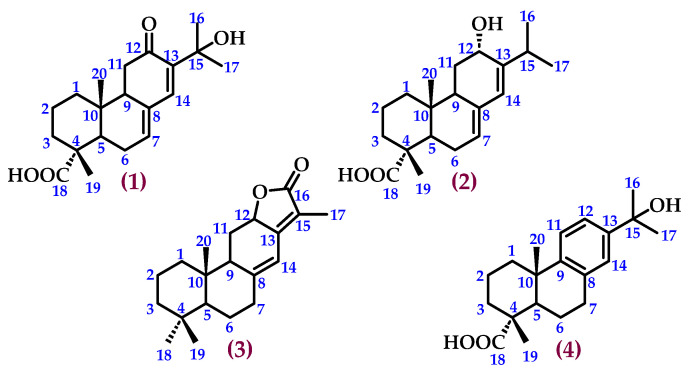
15-Hydroxy-12-oxo-abietic acid (**1**); 12*α*-Hydroxyabietic acid (**2**); (−)-Jolkinolide E (**3**); and 15-Hydroxydehydroabietic acid (**4**), isolated from the dichloromethane/methanol extract of *C. bolivianum*.

Once the compounds were characterised and analysed, they were identified as 15-Hydroxy-12-oxo-abietic acid (**1**); 12*α*-Hydroxyabietic acid (**2**); (−)-Jolkinolide E (**3**); and 15-Hydroxydehydroabietic acid (**4**) (Figure 5).

### 3.2. Viability Assay of the Extracts and Compounds

**Figure 6:**
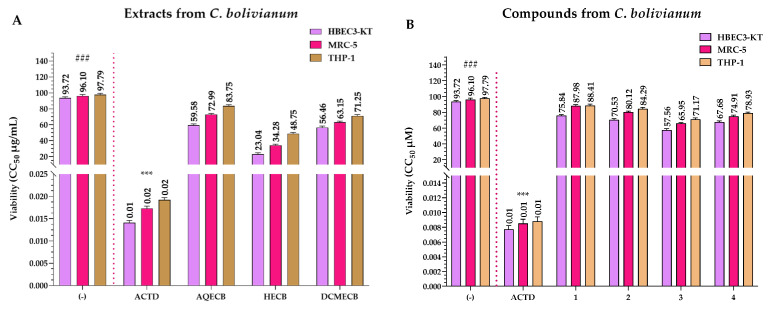
CC_50s_ of the WST-1 (Viability) assays calculated for the extracts (**A**) and compounds (**B**) of *C. bolivianum* at 24 h. (−) = negative control; ACTD = Actinomycin D (positive control); AQECB = aqueous extract of *C. bolivianum*; HECB = *n*-hexane extract of *C. bolivianum*; and DCMECB = dichloromethane/methanol extract of *C. bolivianum*; (###) = Low cytotoxicity and (***) = High cytotoxicity. Viability CC_50_ values are the mean of three independent assays.

Figure 6A shows the viability values of the extracts from *C. bolivianum*, which are higher than that of ACTD. ACTD had CC_50_ values of 0.014 and 0.017 μg/mL for the HBEC3-KT and MRC-5 cell lines, respectively, and 0.019 μg/mL for the THP-1 control cell line.

AQECB exhibited CC_50_ values of 59.58 and 72.99 μg/mL, while DCMECB displayed CC_50_ values of 56.46 and 63.15 μg/mL, respectively. Neither exhibited significant higher cytotoxicity than ACTD in the HBEC3-KT and MRC-5 cell lines. Regarding their cytotoxicity in the THP-1 control cell line, AQECB and DCMECB had CC_50_ values of 83.75 and 71.25 μg/mL, respectively, showing no cytotoxic effects. In contrast, HECB exhibited slightly higher cytotoxicity than AQECB and DCMECB in the HBEC3-KT and MRC-5 cell lines, with CC_50_ values of 23.04 and 34.28 μg/mL, respectively. Furthermore, HECB also demonstrated slight cytotoxicity against the THP-1 control cell line, with a CC_50_ value of 48.75 μg/mL (Figure 6A).

None of the four compounds were as cytotoxic as ACTD, with CC_50_ values of 75.84, 70.53, 57.56, and 67.68 μM for HBEC3-KT, and 87.98, 80.12, 65.95, and 74.91 μM for MRC-5. However, when comparing the compounds with each other, compound **3** showed slightly higher cytotoxicity in both cell lines, followed by compounds **4**, **2**, and **1**. In the case of the control cell line (THP-1), all the compounds were less cytotoxic than ACTD (CC_50_ = 0.0077–0.0088 μM), with CC_50_ values of 88.41, 84.29, 71.17, and 78.93 μM, respectively (Figure 6B).

### 3.3. Anti-Inflammatory Activity of the Extracts and Compounds

In Table 1, the IC_50_ values of the extracts for NF-κB inhibition (stimulated with LPS) can be compared to those of CEL, which had IC_50_ values of 3.24 µg/mL (HBEC3-KT cells), 3.27 µg/mL (MRC-5 cells), and 3.34 µg/mL (THP-1 cells). After analysing the results, we can conclude that DCMECB exhibited higher anti-inflammatory activity than AQECB and DCMECB, as shown by its IC_50_ values of 17.15 µg/mL (HBEC3-KT cells), 23.02 µg/mL (MRC-5 cells), and 43.94 µg/mL (THP-1 cells). 

In Table 1, the IC_50_ values for the NF-κB inhibition (stimulated with LPS) of the compounds can be compared to those of the CEL, with IC_50_ values of 7.15, 7.41, and 7.63 µM in the HBEC3-KT, MRC-5, and THP-1 cell lines, respectively. When analysing the results on the anti-inflammatory activity of the compounds, it was observed that compound **4** exhibited the highest anti-inflammatory activity with IC_50_ values of 10.79, 17.37, and 23.38 µM in the HBEC3-KT, MRC-5, and THP-1 cell lines, respectively.

### 3.4. Antibacterial Activity of the Extracts and Compounds

**Figure 7:**
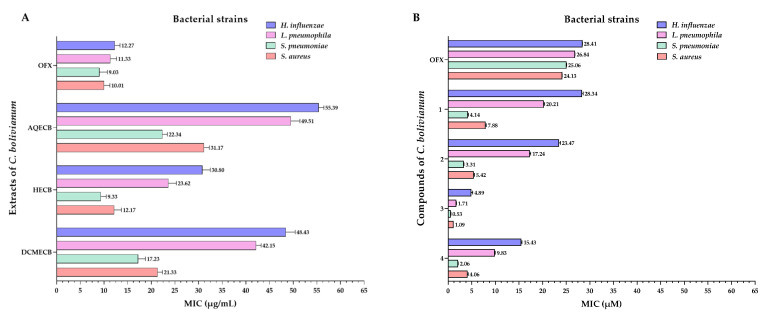
Antibacterial activity of extracts (**A**) and compounds (**B**) on Gram-negative and Gram-positive bacteria at 24 h. OFX = Ofloxacin (positive control); AQECB = aqueous extract of *C. bolivianum*; HECB = *n*-hexane extract of *C. bolivianum*; and DCMECB = dichloromethane/methanol extract of *C. bolivianum*.

All *C. bolivianum* extracts exhibited antibacterial activity against Gram-negative bacteria, but they were not better than those obtained by the OFX, which had MIC values of 12.27 μg/mL for *H. influenzae* and 11.33 μg/mL for *L. pneumophila* (Figure 7A).

In terms of antibacterial activity against Gram-positive bacteria, the HECB was the most active with MIC values of 9.33 μg/mL (*S. pneumoniae*) and 12.17 μg/mL (*S. aureus*). These MIC values were similar to those obtained by the OFX, which were 9.03 μg/mL (*S. pneumoniae*) and 10.01 μg/mL (*S. aureus*). However, this extract was discarded due to its cytotoxicity. Therefore, the DCMECB, which did not present significant cytotoxicity (*p* < 0.001) and had MIC values of 17.23 μg/mL (*S. pneumoniae*) and 21.33 μg/mL (*S. aureus*), was used to carry out the subsequent steps for isolating the compounds (Figure 7A).

Compounds **3** and **4** had MIC values of 4.89, 1.71, 0.53, and 1.09 µM; and 15.43, 9.83, 2.06, and 4.06 µM, respectively, against *H. influenzae*, *L. pneumophila*, *S. pneumoniae*, and *S. aureus*. These values were significantly lower than the OFX (MIC = 24.13–28.41 μM) (*p* < 0.001). In the case of compounds **1** and **2**, although they showed similar activity to the OFX against Gram-negative bacteria, they had higher activity against Gram-positive bacteria (*S. pneumoniae* and *S. aureus*) with MIC values of 4.14; 7.88 µM and 3.31; and 5.42 µM, respectively (Figure 7B).

### 3.5. Anti-Biofilm Activity of the Extracts and Compounds

**Figure 8:**
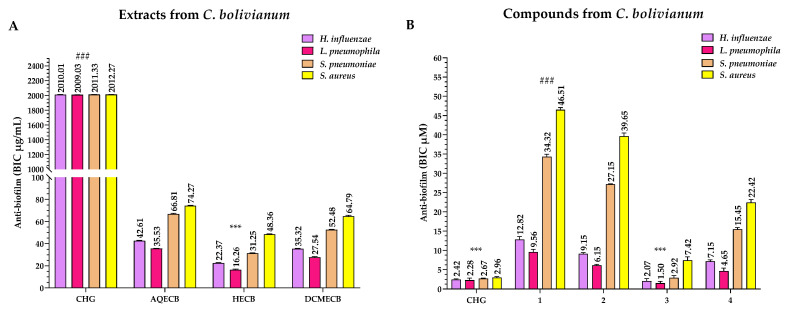
Anti-biofilm activity of extracts (**A**) and compounds (**B**) on Gram-negative and Gram-positive bacteria at 24 h. CHG = Chlorhexidine digluconate (positive control); AQECB = aqueous extract of *C. bolivianum*; HECB = *n*-hexane extract of *C. bolivianum*; and DCMECB = dichloromethane/methanol extract of *C. bolivianum;* (###) = Low antibiofilm capacity and (***) = High antibiofilm capacity.

In Figure 8A, the BIC values of the extracts for biofilm inhibition can be compared with those of CHG, which had BIC values of 2010.01 μg/mL (*H. influenzae*), 2009.03 μg/mL (*L. pneumophila*), 2011.33 μg/mL (*S. pneumoniae*), and 2012.27 μg/mL (*S. aureus*) (Figure 8A). After analysing the results, we can conclude that HECB exhibited higher anti-biofilm activity than AQECB and DCMECB, as demonstrated by its BIC values of 22.37 μg/mL (*H. influenzae*), 16.26 μg/mL (*L. pneumophila*), 31.25 μg/mL (*S. pneumoniae*), and 48.36 μg/mL (*S. aureus*). However, this extract was discarded due to its cytotoxicity. Therefore, the DCMECB extract, with BIC values of 35.32 μg/mL (*H. influenzae*), 27.54 μg/mL (*L. pneumophila*), 52.48 μg/mL (*S. pneumoniae*), and 64.79 μg/mL (*S. aureus*), was used to carry out the subsequent steps for isolating the compounds.

All *C. bolivianum* compounds exhibited anti-biofilm activity against Gram-positive bacteria, but they were not better than those obtained by CHG, which had BIC values of 2.67 μM (*S. pneumoniae*) and 2.96 μM (*S. aureus*). In the case of anti-biofilm activity against Gram-negative bacteria, compound **3** was the most active with BIC values of 2.07 μM (*H. influenzae*) and 1.50 μM (*L. pneumophila*). These BIC values were similar to those obtained by CHG, which were 2.42 μM (*H. influenzae*) and 2.28 (*L. pneumophila*) μM, respectively (Figure 8B).

### 3.6. Anti-Quorum Sensing Activity of the Extracts and Compounds

The extracts from *C. bolivianum* demonstrated anti-quorum sensing activity; however, they were less effective compared to Furanone C-30, which showed Anti-QS values of 3.21 μg/mL for *H. influenzae*, 3.03 μg/mL for *L. pneumophila*, 3.44 μg/mL for *S. pneumoniae*, and 3.77 μg/mL for *S. aureus* (Table 2). Additionally, HECB was discarded due to its cytotoxicity.

Unlike the extracts of *C. bolivianum*, the compounds of *C. bolivianum* had lower Anti-QS values than Furanone C-30, with compound **3** being the most active, showing Anti-QS values of 0.64 µM, 0.31 µM, 3.01 µM, and 4.88 µM against *H. influenzae*, *L. pneumophila*, *S. pneumoniae*, and *S. aureus*, respectively (Table 2). It is noteworthy that the compounds exhibited higher activity against Gram-negative bacteria. This may be attributed to the compounds potentially inhibiting QS activity mediated by acyl-HSL. To confirm this hypothesis, a quantitative violacein inhibition assay was conducted on the *C. violaceum* strain.

### 3.7. Inhibition of Violacein by Compounds of C. bolivianum

The results on the inhibition capacity of violacein production regulated by QS against *C. violaceum* (based on the measurement of the inhibition radius of the pigment in mm) are presented in Table 3. As shown below, compound **3** had a higher activity (Anti-QS) level than the CIP (1.51 µM).

At 0.94 µM, compound **3** presented a 78.13% inhibition of violacein, while compound **4** showed a 71.60% inhibition at a concentration of 1.16 µM (Table 3).

## 4. Discussion

To create a ^1^H NMR fingerprint of the *C. bolivianum* species (Figure S6 and Table S1), a literature review of the known compounds was conducted. Among the compounds reported by other authors, most were volatile oils from the monoterpene family (acyclic and cyclic, with an ester fragment in their structures) and sesquiterpenes (monocyclic and bicyclic) [52], a phenolic acid (in glucoside and aglycone form), and flavonoids, including their glycosylated derivatives [53]. Using this information, each compound was assigned a specific signal in the experimental ^1^H NMR spectra (Figure S6).

In our case, a detailed analysis of the NMR spectra of the extracts revealed that the ^1^H NMR spectrum of HECB shows that almost all the signals correspond to non-polar compounds, such as sesquiterpenoids, which can be seen in the initial ranges of the spectrum (Figures S2 and S3). Additionally, in the ^1^H NMR spectrum of DCMECB, characteristic signals of diterpenes with abietane and dehydroabietane structures could be observed. Typical signals of diterpenes with a lactone ring were also detected (Figures S4 and S5).

Regarding the compounds, our research reports for the first time the isolation of compounds **1**, **2**, **3**, and **4** from the AQECB of *C. bolivianum*. However, previous research has already identified these compounds in other plant species. For instance, compound **1** was isolated and characterised in *Pinus koraiensis* [54]. Compound **2** was isolated and characterised in *Euphorbia calyptrata* [55]. Similarly, compound **3** was isolated and characterised in *Pseudolarix amabilis* [56]. Finally, compound **4** was isolated and characterised in *Pinus massoniana* [43].

The cytotoxicity of extracts from species of the *Clinopodium* genus has been the subject of several relevant studies. For example, it has been reported that aqueous extracts of *C. tomentosum* and *C. gracile* show no cytotoxic effects at concentrations below 50 μg/mL and 10 mg/mL, respectively [57,58]. Furthermore, in vivo studies have demonstrated that aqueous extracts of *C. vulgare* L. and *C. bolivianum* have a median lethal dose higher than 2000 mg/kg [59,60], suggesting that these extracts are safe at therapeutic doses.

In our investigation into the cell viability of *C. bolivianum* extracts (Figure 6A), none showed significant cytotoxicity in the HBEC3-KT, MRC-5, and THP-1 cell lines compared to ACTD, which exhibited cytotoxicity a thousand times higher than the most cytotoxic C. bolivianum extract, HECB. These findings align with Mohanty et al. [53], who found that at a dose of 400 μg/mL of ethanolic extract from *C. bolivianum*, bladder cells showed an average survival rate of 90%.

Additionally, our studies indicate that the organic extract HECB had the highest cytotoxicity, possibly due to its lipophilic compounds, which have higher permeability and can accumulate at higher concentrations, leading to cellular cytotoxicity [61]. These findings support those reported by Solis et al. [59], who demonstrated the cytotoxic effects of organic extracts of *C. bolivianum* (essential oil extract) at doses of 1000 μg/mL and a median lethal concentration (DC_50_) of 737.58 μg/mL.

Regarding the cytotoxicity of the isolated compounds (Figure 6B), none showed significant cytotoxicity compared to ACTD, with CC_50_ values above 55 μM for all compounds. However, compound **3** showed slight cytotoxicity, followed by compounds **4**, **2**, and **1** when compared to each other. Previous studies reported that compound **3** has a cytotoxicity of 37.23 and 39.53 μM in Hep3B and Huh7 cells, respectively [45]. These data differ from our findings, where we reported CC_50_ concentrations > 70 μM, possibly due to differences in the tested cell lines. Furthermore, Michavila et al. [50] reported that chemical compounds derived from dehydroabietic acid showed no cytotoxicity at CC_50_ values above 80 μM, confirming our data for compounds **1**, **3**, and **4**, whose CC_50_ values are in a similar range due to their shared chemical structures (core).

However, to delve deeper into the causes of the different cytotoxicity values, the lipophilicity of the compounds was studied. Previous studies have reported that cytotoxicity is closely correlated with lipophilicity, as a higher lipophilicity allows for a higher accumulation of compounds within cells, leading to more pronounced toxic effects [62,63]. In our study, it was observed that compound **1** has a LogP of 2.58, followed by compound **2** (LogP: 3.25), compound **4** (LogP: 3.32), and finally, compound **3**, which exhibits the highest lipophilic properties with a LogP of 4.90 [64]. Thus, it can be concluded that the difference in cytotoxicity levels between the compounds is largely due to their varying degrees of lipophilicity.

Although the cytotoxicity results of our study did not show significant effects in the different cell lines, it is important to consider that cytotoxicity results can vary significantly between different cell types due to factors such as receptor expression, compound penetration ability, and inherent cell sensitivity [65]. Comparing our results with previous studies [45,50,53,57,58,59,60,61], we can highlight the importance of selecting appropriate cell lines (cells from tissues or organs that are affected by the diseases being researched) to accurately assess cytotoxicity. Furthermore, the lack of significant effects in the various cell lines could indicate that the compounds have a relatively high safety profile in these specific cells, although they may have different effects in other cell types or at different doses.

Regarding the potential side effects of compound **3**, it is crucial to conduct further studies to evaluate its complete toxicity profile. Current data suggest that compound **3** has slight cytotoxicity in the various cell lines, but additional research is needed to better understand its potential adverse effects in different biological contexts. 

Finally, we should note that this is the first report on cell viability for compounds **1**, **2**, and **4**, and our findings contribute to a broader understanding of the cytotoxicity and lipophilic properties of these compounds, which may inform future studies on their therapeutic potential and safety.

Various species of the *Clinopodium* genus have long been the focus of studies looking into their anti-inflammatory properties. Burk et al. [66] reported that the aqueous extract of *C. vulgare* reduced PGE2 levels at a concentration of 300 μg/mL by approximately 20% through the direct inhibition of COX-2 activity in RAW264.7 cells. Additionally, Amirova et al. [67] found that the aqueous-methanol extract of *C. vulgare* significantly inhibited COX-2 production in neutrophils at a concentration of 1 μg/mL. Moreover, Wang et al. [68] indicated that the 70% ethanol extract of *C. chinense* inhibited NF-κB activation in RAW264.7 cells at a concentration of 50 μg/mL.

In our investigation of *C. bolivianum*, we evaluated the aqueous (AQECB), *n*-hexane (HECB), and dichloromethane/methanol (DCMECB) extracts to determine their ability to inhibit NF-κB in HBEC3-KT, MRC-5, and THP-1 cells. The IC_50_ values obtained from the extracts indicate that DCMECB was the most effective in inhibiting NF-κB, with values of 17.15 μg/mL (HBEC3-KT) and 23.02 μg/mL (MRC-5), showing potent anti-inflammatory activity.

Comparing our results with previous studies, we observed that hydroethanolic extracts of *C. bolivianum* significantly reduced IL-8 levels in urothelial cells at concentrations of 400 μg/mL [53]. Furthermore, Apaza et al. [69] evaluated the anti-inflammatory activity (NF-κB inhibition) of *C. bolivianum* extracts, finding that the dichloromethane/methanol extract had IC_50_ values of 38.88 μg/mL (MRC-5 cells) and 42.81 μg/mL (HBEC3-KT cells), being more active than aqueous and *n*-hexane extracts.

These findings suggest that extracts from various *Clinopodium* species have shown anti-inflammatory potential through different mechanisms. These include inhibition of the NF-κB pathway, crucial in regulating pro-inflammatory genes, by inhibiting IκB*α* phosphorylation and/or reducing MAPK activation [70]. Additionally, significant suppression of inflammatory mediators such as IL-8 and NO has been observed, indicating a direct modulation of cellular inflammatory response [71]. All these inhibitory mechanisms are attributed to hydroxylated aromatic compounds, such as caffeic acid and chlorogenic acid, identified as being responsible for inhibiting enzymes like COX-2, which are essential in the synthesis of pro-inflammatory prostaglandins.

In relation to the anti-inflammatory activity of the compounds, it has been previously reported that compound **4** inhibits nitric oxide (NO) production in RAW 264.7 cells, with an IC_50_ of 50.9 µM [72]. In our study, we found that this activity is related to the inhibition of NF-κB activation. By comparing both studies, it can be suggested that the inhibition of NF-κB could reduce the expression of iNOS and, consequently, the production of NO. NF-κB increases NO production in inflammatory cells by activating the transcription of iNOS, which amplifies the inflammatory response by inducing the synthesis of pro-inflammatory cytokines and other mediators [73]. Thus, NF-κB directly regulates inflammation by promoting cytokine expression and influences NO production through iNOS activation.

To understand how compound **4** inhibits NF-κB activation, it is essential to examine its structural characteristics and compare them with Celastrol, a known NF-κB inhibitor. Compound **4** has a typical diterpenoid core of abietic acid, a tricyclic structure that confers rigidity and has a suitable framework for specific protein interactions. The presence of a hydroxyl group at position C15 and a ketone group at position C12 introduces polar features that facilitate hydrogen bonding and interaction with polar or charged residues in target proteins, which is key to its biological activity [74]. This polar structure has been observed in other oxime-type derivatives of the diterpene dehydroabietinol, which also showed significant anti-inflammatory activity (with IC_50_ between 13.8 and 18.7 μM) by reducing IL-1*β* and caspase-1 expression [75].

Celastrol, on the other hand, is a triterpenoid with a quinone core and several reactive functional groups, including carbonyl groups and a quinone system. These groups allow Celastrol to form covalent bonds with cysteine residues in the active site of IKK*β*, an essential kinase in the NF-κB activation pathway. The formation of these bonds inhibits IKK*β* activity, preventing the phosphorylation and subsequent degradation of IκB*α*, thus retaining NF-κB in the cytoplasm [76]. Although compound **4** does not have a quinone system to form covalent bonds similarly, its ketone group at C12 and hydroxyl group at C15 could interact with IKK*β* through hydrogen bonds and Van der Waals forces. These non-covalent interactions could stabilise an inactive conformation of IKK*β*, thereby blocking NF-κB activation in a manner analogous to Celastrol, albeit via a different interaction mechanism. Additionally, abietane-type diterpenoid diepoxide compounds, such as triptolide, have also shown anti-inflammatory activity by inhibiting the classical NF-κB and MAPK pathways [75].

Besides inhibiting the phosphorylation of NF-κB subunits (such as p65) and proteins (such as IκB), it is crucial to prevent their translocation to the nucleus and their transcriptional activity [77]. Celastrol achieves this by modifying the conformation of NF-κB, indirectly reducing its capacity to be phosphorylated and translocated [78]. Similarly, compound **4** could alter the conformation of NF-κB through polar interactions, reducing its affinity for the kinase proteins responsible for its activation and translocation.

Once in the nucleus, NF-κB must bind to DNA to activate the transcription of inflammatory genes [79]. Celastrol interferes with this binding by modifying the structure of NF-κB, decreasing its DNA affinity [80]. Although compound **4** does not form covalent bonds, it could alter the tertiary structure of NF-κB through its polar functional groups, effectively preventing DNA binding and reducing the transcription of inflammatory genes.

Although the other compounds also demonstrated anti-inflammatory activity, it was less significant compared to compound **4** and the positive control, Celastrol. In this study, we focused exclusively on analysing the potential anti-inflammatory mechanisms of the most active compound, without delving into the mechanisms of action of the other compounds.

Previous research has reported the antibacterial activity of other species within the *Clinopodium* genus. For instance, the essential oil of *C. brownei* demonstrated antibacterial activity against Gram-negative strains such as *E. coli* (MIC = 6.22 mg/mL), *P. vulgaris* (MIC = 4.62 mg/mL), *Klebsiella oxytoca* (*K. oxytoca*, MIC = 7.18 mg/mL), and *P. aeruginosa* (MIC = 8.38 mg/mL), as well as against Gram-positive strains such as *S. aureus* (MIC = 7.92 mg/mL), *Enterococcus faecalis* (*E. faecalis*, MIC = 5.54 mg/mL), *L. grayi* (MIC = 3.84 mg/mL), and *Staphylococcus epidermidis* (*S. epidermidis*, MIC = 13.57 mg/mL) [22]. Similarly, Noriega et al. [23] reported the antibacterial activity of the essential oil of *C. nubigenum* against *S. mutans*, *S. pyogenes*, and *S. pneumoniae* at a concentration of 0.6% *v*/*v*.

Moreover, the acetone and ethyl acetate extracts of *C. vulgare* exhibited antibacterial activity with MICs ranging from 625 to 10 mg/mL and from 0.625 to 20 mg/mL, respectively, against bacterial strains such as *E. coli*, *S. aureus*, *P. aeruginosa*, *Bacillus subtilis* (*B. subtilis*), *E. faecalis*, and *Klebsiella pneumoniae* (*K. pneumoniae*) [81]. Antibacterial activity was also reported for the *n*-hexane (MIC = 500 μg/mL), methanol (MIC = 250 μg/mL), and ethyl acetate (MIC = 1000 μg/mL) extracts of *C. taxifolium* against *K. pneumoniae* and *P. aeruginosa* [82]. Finally, the methanolic extract of *C. nepeta* showed antibacterial activity against strains of *E. coli*, *P. aeruginosa*, *Bacillus cereus* (*B. cereus*), and *K. pneumoniae* at a concentration ranging from 2.5 to 15 μg/mL [83].

Based on these preliminary studies, it can be stated that species of the *Clinopodium* genus exhibit antibacterial activity. Additionally, our study reported that extracts of *C. bolivianum* showed higher activity against Gram-positive bacterial strains (*S. pneumoniae* and *S. aureus*) at lower concentrations than those reported for other species in the *Clinopodium* genus. The most active extracts were HECB and DCMECB, although the former was discarded due to its high cytotoxicity. Therefore, the DCMECB was fractionated to isolate and identify compounds with antibacterial activity.

Among the compounds isolated from DCMECB, compounds **3** and **4** showed higher activity than compounds **1** and **2** and the positive control. Compound **3**, with a sesquiterpene lactone structure, acts through multiple pathways: it interacts with the phospholipids of the bacterial cell membrane, causing disruptions in its integrity and function, leading to bacterial lysis; it inhibits bacterial protein synthesis by interfering with ribosomes or translation processes; and it modulates internal metabolic processes, generating cellular stress that leads to bacterial death [84,85,86].

The mechanism of compound **4** (a derivative of dehydroabietic acid) involves specific interactions with the bacterial cell membrane, facilitated by the hydroxyl group at position 15, compromising membrane integrity and causing the release of essential cellular components. Additionally, it inhibits key enzymes involved in bacterial cell wall synthesis or essential biosynthetic processes, affecting bacterial structure and functionality [87].

The difference in the antibacterial activity of these compounds between Gram-negative and Gram-positive bacteria is explained by the structural differences in their cell walls. Gram-negative bacteria, with a more complex cell wall structure that includes an outer membrane of lipopolysaccharides, may be less susceptible due to the additional barrier that hinders the penetration of hydrophobic compounds like **3** and **4** [88]. In contrast, Gram-positive bacteria, with a simpler cell wall structure mainly composed of peptidoglycan, are more permeable to these compounds, reflected in generally lower MIC values [89].

It is important to mention that anti-biofilm (BIC) and anti-quorum sensing (Anti-QS) activities are closely related. Biofilms are formed by bacteria embedded in an exopolysaccharide matrix, constituting a major resistance mechanism by preventing antibiotics from penetrating to exert their action. The formation and maturation of these biofilms are due to the quorum sensing process, which consists of bacterial communication through autoinducers [90]. To understand the differences in Anti-QS activity between the compounds evaluated against Gram-negative and Gram-positive bacteria, it is crucial to consider the types of autoinducers used by these microorganisms. Gram-negative bacteria, such as *H. influenzae* and *L. pneumophila*, typically employ *N*-acyl homoserine lactones (AHLs), while Gram-positive bacteria, such as *S. pneumoniae* and *S. aureus*, mainly use cyclic oligopeptides [91].

Although previous information is limited, there is evidence demonstrating the anti-biofilm activity of the hydroethanolic extract of *C. bolivianum* at a concentration of 400 μg/mL against uropathogenic strains of *E. coli* [53]. This reinforces our study on the anti-biofilm potential of species within the *Clinopodium* genus. In our case, the extracts showed higher anti-biofilm activity against Gram-negative bacteria at concentrations six times lower than those reported by Mohanty et al. [53], with DCMECB being the most active.

In the case of the compounds, compounds **3** and **4** had very significant results regarding their anti-biofilm capacity. Compound **3** is a sesquiterpene lactone that shows a high anti-biofilm efficacy against a variety of Gram-negative and Gram-positive bacteria. The structure of compound **3** includes a lactone ring, which functionally resembles the AHLs used by many Gram-negative bacteria. This structural similarity suggests that compound **3** could act as an AHL analogue, effectively competing for autoinducer receptors in bacteria such as *H. influenzae* and *L. pneumophila*. This would explain the significantly lower BIC and Anti-QS values observed for compound **3** in these bacteria, compared to Gram-positive bacteria (*S. pneumoniae* and *S. aureus*), which use different autoinducers (small cyclic peptides) [92,93].

Additionally, the functional groups present in compound **3**, such as the carbonyl groups in the lactone, can interact with bacterial proteins and enzymes, inhibiting their function and contributing to biofilm destabilisation. These interactions can induce oxidative stress, damaging critical cellular components and leading to biofilm disintegration [94]. Another mechanism of action of compound **3** is the inhibition of bacterial adhesion to surfaces, which is an essential step in the initial biofilm formation. This compound can alter the expression of genes responsible for the production of adhesins, and surface proteins necessary for the initial adherence of bacteria to surfaces [84].

The anti-biofilm activity of compound **4** may be due to the hydroxyl group at position 15, which plays a crucial role in interacting with AHL receptors in Gram-negative bacteria [95]. However, the BIC and Anti-QS values were higher in Gram-positive bacteria (*S. pneumoniae* and *S. aureus*), suggesting that compound **4** may be less effective against the small cyclic peptides used by these bacteria. The hydroxyl groups can form hydrogen bonds with components of the biofilm’s extracellular matrix, destabilising it and facilitating the compound’s penetration [96]. The rigid and flat structure of the abietic nucleus allows for effective interaction with bacterial membranes, destabilising them and increasing their permeability, leading to cell lysis [97]. This action is particularly effective against Gram-negative bacteria. Additionally, compound **4** may inhibit the synthesis of the biofilm’s extracellular matrix, reducing the protection this matrix provides to bacteria embedded in the biofilm. The presence of the hydroxyl group can also induce an imbalance in bacterial redox systems, generating ROS that damage proteins, lipids, and nucleic acids within bacterial cells, with this oxidative stress being particularly effective against Gram-positive bacteria [84,87].

The anti-biofilm efficacy of compounds **3** and **4** varies between Gram-negative and Gram-positive bacteria due to the structural and compositional differences in their cell walls and extracellular matrices. Gram-negative bacteria, such as *H. influenzae* and *L. pneumophila*, possess an additional outer membrane that can hinder the penetration of anti-biofilm agents. However, compound **3** shows a notable capacity to inhibit quorum sensing and disintegrate the extracellular matrix, which is crucial for overcoming this barrier [98]. Compound **4**, on the other hand, seems effective in destabilising the outer membrane and inhibiting matrix synthesis [99].

In contrast, Gram-positive bacteria, such as *S. pneumoniae* and *S. aureus*, have a thicker cell wall but lack the outer membrane. This makes them more susceptible to compounds that induce oxidative stress and destabilise the extracellular matrix. Both compound **3** and compound **4** show strong activity against these bacteria, with compound **3** generating oxidative stress, and compound **4** interfering with bacterial redox systems.

To evaluate the effect of the compounds on quorum sensing in Gram-negative bacteria, a violacein assay with *C. violaceum* was conducted. The results showed that compound **3** exhibited significant antimicrobial activity, with BIC values of 1.62 µM and Anti-QS values of 0.94 µM. These values suggest that compound **3** is highly effective in inhibiting both biofilm formation and quorum sensing in this bacterial strain.

The structural similarity between compound **3** and AHLs (five-membered lactone ring attached to an acyl chain) suggests that the compound might act as an antagonist of quorum sensing signals [100]. This implies that compound **3** could interfere with the bacterial signalling system in a manner similar to quorum sensing inhibitors, disrupting intercellular communication and thereby reducing bacterial virulence [84].

This interaction with the quorum sensing system highlights the potential of compound **3** as an Anti-QS agent, offering a new avenue for developing treatments against bacterial infections based on quorum sensing.

Thus, our study has confirmed the previously reported therapeutic properties of *C. bolivianum*. The results support the effectiveness of this plant in modulating inflammation and in antibacterial activity, corroborating earlier findings regarding its therapeutic potential. This confirmation not only validates the traditional use of the plant but also reinforces its relevance in the context of respiratory diseases, which continue to be a significant challenge for public health.

Moreover, the added value of our research lies in the identification of a new class of bioactive compounds in this plant species (diterpenes). Previous studies had focused on monoterpenes and sesquiterpenes, which are known for their beneficial properties. Our research expands this knowledge by revealing the presence of diterpenes, which also exhibit significant anti-inflammatory and antibacterial properties. This finding represents a significant advance in the phytochemical profile of *C. bolivianum* and suggests that diterpenes may play a crucial role in the overall therapeutic efficacy of the plant.

The confirmation of the previously described properties and the identification of diterpenes as new active compounds highlight the importance of conducting further studies to thoroughly explore the therapeutic potential of *C. bolivianum*. In this regard, it is essential to carry out clinical trials to validate the safety and efficacy of extracts containing diterpenes in the treatment of respiratory diseases.

Additionally, future research should focus on evaluating the combination of these diterpenes to optimise therapeutic outcomes through potential synergies. Finally, optimising the activity of diterpenes through structural modifications and structure–activity relationship (SAR) studies can further enhance their therapeutic profile, increasing the efficacy and reducing toxicity.

## 5. Conclusions

This study is the first to document the isolation of diterpenoid-like compounds from *C. bolivianum*. Although these compounds have previously been identified in other plant species, their identification in *C. bolivianum* is crucial for establishing a phytochemical profile that can be compared with other species in the *Clinopodium* genus. The findings underscore the therapeutic potential of *C. bolivianum* in anti-inflammatory and antibacterial activities. The dichloromethane/methanol extract (DCMECB) showed a potent inhibition of NF-κB activation in HBEC3-KT and MRC-5 cells, suggesting notable anti-inflammatory activity. Among the isolated compounds, (−)-Jolkinolide E (**3**) and 15-Hydroxydehydroabietic acid (**4**) showed significant anti-inflammatory activity.

In terms of antibacterial activity, the HECB and DCMECB extracts were effective against Gram-positive bacteria such as *S. pneumoniae* and *S. aureus*. Compounds **3** and **4** also exhibited remarkable antibacterial activity. Furthermore, the extracts and compounds from *C. bolivianum* demonstrated anti-biofilm and anti-quorum sensing activities, being more effective against Gram-negative bacteria. Compound **3** showed efficacy in interfering with quorum sensing processes in Gram-negative bacteria.

In conclusion, our study not only validates the traditional therapeutic properties of *C. bolivianum*, but also highlights the discovery of diterpenes as a valuable addition to the plant’s phytochemical profile. This discovery opens up new research opportunities and underscores the potential of *C. bolivianum* in developing new therapies for bacterial respiratory diseases.

## Figures and Tables

**Table 1:** IC_50s_ of the inhibition of NF-κB activation, calculated for the samples (extracts and compounds of *C. bolivianum*). NF-κB IC_50_ values are the mean of three independent assays.

Samples No.	NF-κB Inhibition at 24 h IC_50_ ± SEM (μM) ^a^		
	HBEC3-KT	MRC-5	THP-1
AQECB (*)	37.36 ± 0.52	44.95 ± 0.94	55.74 ± 0.18
HECB (*)	51.12 ± 0.67	57.38 ± 0.77	70.62 ± 0.88
DCMECB (*)	17.15 ± 0.28	23.02 ± 0.55	43.94 ± 0.74
CEL (*)	3.24 ± 0.02	3.27 ± 0.04	3.34 ± 0.07
1	27.94 ± 0.89	39.01 ± 0.22	43.38 ± 0.73
2	21.95 ± 0.23	26.94 ± 0.61	32.61 ± 0.69
3	17.98 ± 0.37	23.96 ± 0.21	29.45 ± 0.48
4	10.79 ± 0.69	17.37 ± 0.82	23.38 ± 0.71
CEL	7.15 ± 0.85	7.41 ± 0.83	7.63 ± 0.86

(*) = μg/mL. (μM) ^a^, where “a” indicates that the concentration units in μM apply only to the compounds. CEL = Celastrol (positive control); AQECB = aqueous extract of *C. bolivianum*; HECB = *n*-hexane extract of *C. bolivianum*; and DCMECB = dichloromethane/methanol extract of *C. bolivianum.*

**Table 2:** Inhibition of quorum sensing, calculated for the samples (extracts and compounds of *C. bolivianum*) on Gram-negative and Gram-positive bacteria at 24 h. Anti-QS values are the mean of three independent assays.

Samples No.	Quorum Sensing Inhibition at 24 h Anti-QS ± SEM (μM) ^a^			
	*H. influenzae*	*L. pneumophila*	*S. pneumoniae*	*S. aureus*
AQECB (*)	23.98 ± 0.07	17.18 ± 0.08	38.08 ± 0.02	42.61 ± 0.04
HECB (*)	9.36 ± 0.05	7.18 ± 0.09	18.17 ± 0.05	23.69 ± 0.09
DCMECB (*)	16.41 ± 0.04	13.25 ± 0.03	32.42 ± 0.08	37.25 ± 0.09
Furanone C-30 (*)	3.21 ± 0.02	3.03 ± 0.08	3.44 ± 0.06	3.77 ± 0.04
1	4.64 ± 0.09	2.44 ± 0.09	9.89 ± 0.04	12.24 ± 0.08
2	3.19 ± 0.04	1.95 ± 0.07	8.14 ± 0.05	11.81 ± 0.07
3	0.64 ± 0.01	0.31 ± 0.03	3.01 ± 0.07	4.88 ± 0.09
4	2.39 ± 0.03	1.21 ± 0.04	7.78 ± 0.01	10.08 ± 0.08
Furanone C-30	11.81 ± 0.05	11.59 ± 0.03	12.03 ± 0.08	12.67 ± 0.02

(*) = μg/mL. (μM) ^a^, where “a” indicates that the concentration units in μM apply only to the compounds. Furanone C-30 (positive control); AQECB = aqueous extract of *C. bolivianum*; HECB = *n*-hexane extract of *C. bolivianum*; and DCMECB = dichloromethane/methanol extract of *C. bolivianum.*

**Table 3:** Anti-biofilm and anti-quorum sensing activities of compounds from C*. bolivianum* on *C. violaceum*.

Samples No.	*C. violaceum*		
	BIC (µM)	Anti-QS (µM)	% of Violacein Inhibition
1	11.31 ± 0.07	4.83 ± 0.05	20.45
2	7.54 ± 0.05	3.55 ± 0.06	48.88
3	1.62 ± 0.06	0.94 ± 0.03	78.13
4	5.81 ± 0.02	1.16 ± 0.06	71.60
CIP	2.13 ± 0.01	1.51 ± 0.01	71.68

CIP = Ciprofloxacin hydrochloride (positive control).

## Data Availability

The datasets and materials used and/or analysed during the current study are available from the corresponding author upon reasonable request.

## Supplementary Materials

The following supporting information can be downloaded at: https://www.mdpi.com/article/10.3390/pharmaceutics16081094/s1. The spectra (NMR) for the extract and compounds from *C. bolivianum* tested in this study are provided as supporting information (Figures S1–S26). Marker compounds of *C. bolivianum* identified in the 1H NMR spectra of the different extracts (Table S1).
