# Supplementary material for: Anti-Inflammatory, Antibacterial, Anti-Biofilm, and Anti-Quorum Sensing Activities of the Diterpenes Isolated from Clinopodium bolivianum
Source: Pharmaceutics. 2024 Aug 20;16(8):1094. doi: 10.3390/pharmaceutics16081094 (PMC11360483; doi:10.3390/pharmaceutics16081094)
Supplement: Supplementary file 1 [file pharmaceutics-16-01094-s001.zip › pharmaceutics-3145585-supplementary.pdf]

Supplementary Materials

Anti-Inflammatory, Antibacterial, Anti-Biofilm and Anti-Quorum Sensing Activities of the Diterpenes Isolated from *Clinopodium bolivianum*

Luis Apaza Ticona, Ana Martínez Noguerón, Javier Sánchez Sánchez-Corral, Natalia Montoto Lozano, Monserrat Ortega Domenech

Contents:

- **Figure S1**. ^{1}H NMR spectrum of AQECB in D_2O 300 MHz.
- **Figure S2**. ^{1}H NMR spectrum of HECB in $CDCl_3$ 300 MHz.
- **Figure S3**. ^{13}C NMR spectrum of HECB in $CDCl_3$ 75 MHz.
- **Figure S4**. ^{1}H NMR spectrum of DMECB in $CDCl_3$ 300 MHz.
- **Figure S5**. ^{13}C NMR spectrum of DMECB in $CDCl_3$ 75 MHz.
- **Figure S6**. Comparison of the ^{1}H NMR signals of the compounds present in the different extracts of *Clinopodium bolivianum* in the range of 0.0 to 9.0 ppm at 300 MHz. The symbols in the spectra correspond to the resonances of the main groups of marker compounds.
- **Figure S7**. ^{1}H NMR spectrum of 15-Hydroxy-12-oxo-abietic acid (**1**) in $CDCl_3$ 300 MHz.
- **Figure S8**. ^{13}C NMR spectrum of 15-Hydroxy-12-oxo-abietic acid (**1**) in $CDCl_3$ 75 MHz.
- **Figure S9**. DEPT-135 spectrum of 15-Hydroxy-12-oxo-abietic acid (**1**) in $CDCl_3$ 75 MHz.
- **Figure S10**. ^{1}H-^{1}H COSY spectrum of 15-Hydroxy-12-oxo-abietic acid (**1**) in $CDCl_3$.
- **Figure S11**. ^{1}H-^{13}C HSQC spectrum of 15-Hydroxy-12-oxo-abietic acid (**1**) in $CDCl_3$.
- **Figure S12**. ^{1}H-^{13}C HMBC spectrum of 15-Hydroxy-12-oxo-abietic acid (**1**) in $CDCl_3$.
- **Figure S13**. ^{1}H NMR spectrum of 12α-Hydroxy-abietic acid (**2**) in $CDCl_3$ 300 MHz.
- **Figure S14**. ^{13}C NMR spectrum of 12α-Hydroxy-abietic acid (**2**) in $CDCl_3$ 75 MHz.
- **Figure S15**. DEPT-135 spectrum of 12α-Hydroxy-abietic acid (**2**) in $CDCl_3$ 75 MHz.
- **Figure S16**. ^{1}H-^{1}H COSY spectrum of 12α-Hydroxy-abietic acid (**2**) in $CDCl_3$.
- **Figure S17**. ^{1}H-^{13}C HSQC spectrum of 12α-Hydroxy-abietic acid (**2**) in $CDCl_3$.
- **Figure S18**. ^{1}H-^{13}C HMBC spectrum of 12α-Hydroxy-abietic acid (**2**) in $CDCl_3$.
- **Figure S19**. ^{1}H NMR spectrum of (-)-Jolkinolide E (**3**) in $CDCl_3$ 300 MHz.
- **Figure S20**. ^{13}C NMR spectrum of (-)-Jolkinolide E (**3**) in $CDCl_3$ 75 MHz.
- **Figure S21**. ^{1}H NMR spectrum of 15-Hydroxy-dehydroabietic acid (**4**) in $CDCl_3$ 300 MHz.
- **Figure S22**. ^{13}C NMR spectrum of 15-Hydroxy-dehydroabietic acid (**4**) in $CDCl_3$ 75 MHz.
- **Figure S23**. DEPT-135 spectrum of 15-Hydroxy-dehydroabietic acid (**4**) in $CDCl_3$ 75 MHz.
- **Figure S24**. ^{1}H-^{1}H COSY spectrum of 15-Hydroxy-dehydroabietic acid (**4**) in $CDCl_3$.
- **Figure S25**. ^{1}H-^{13}C HSQC spectrum of 15-Hydroxy-dehydroabietic acid (**4**) in $CDCl_3$.
- **Figure S26**. ^{1}H-^{13}C HMBC spectrum of 15-Hydroxy-dehydroabietic acid (**4**) in $CDCl_3$.
- **Table S1**. Marker compounds of *C. bolivianum* identified in the ^{1}H NMR spectra of the different extracts.

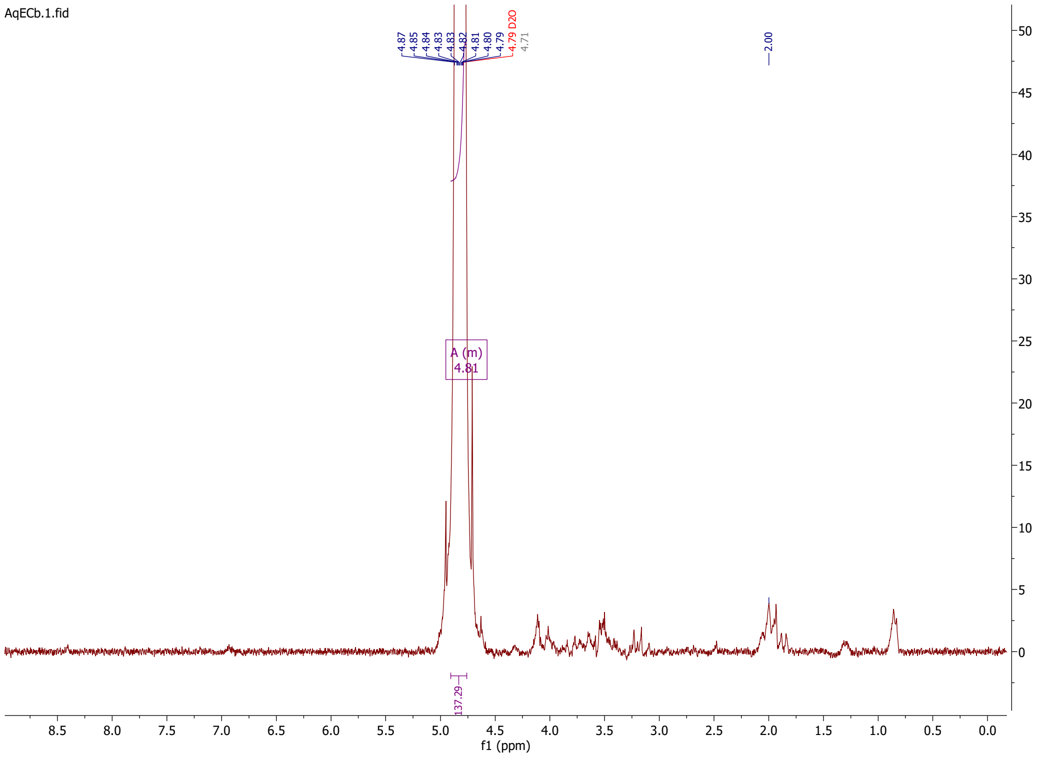

- **Figure S1**. ^{1}H NMR spectrum of AQECB in D_2O 300 MHz.

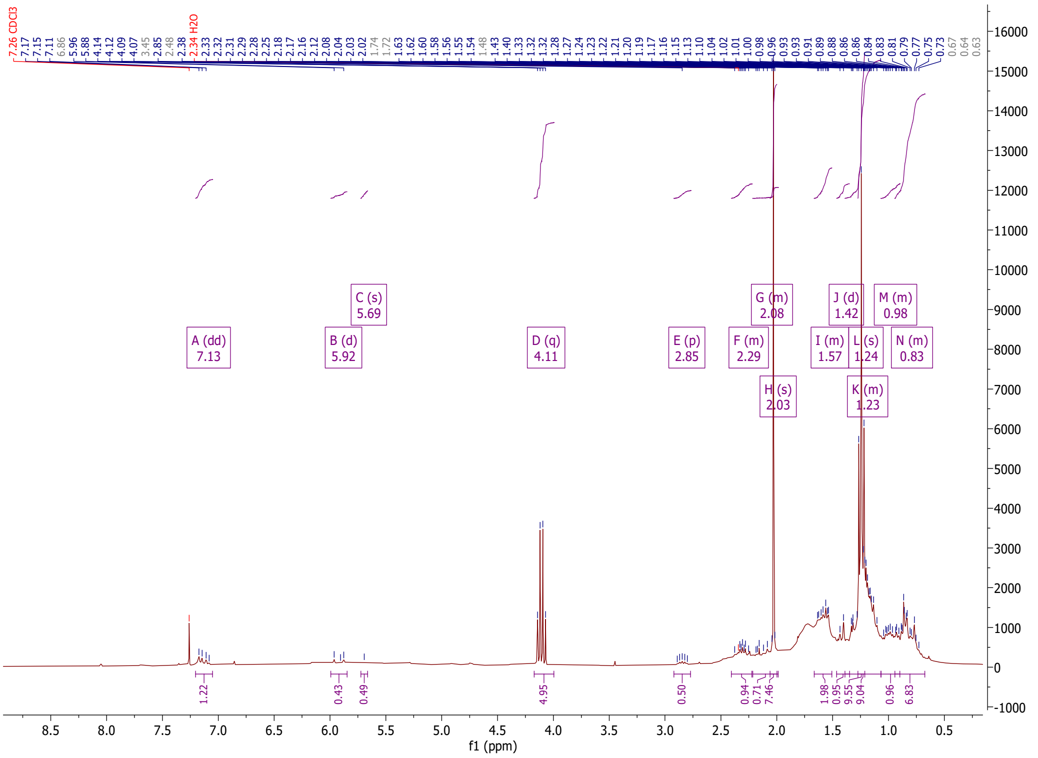

- **Figure S2**. ^{1}H NMR spectrum of HECB in $CDCl_3$ 300 MHz.

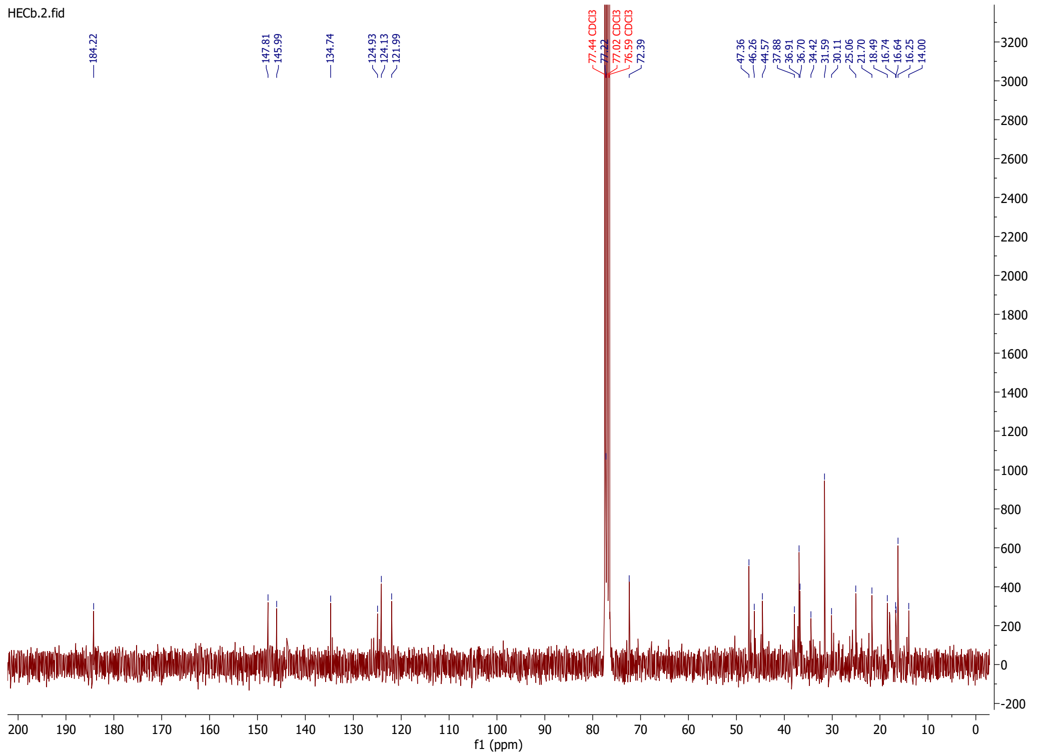

- **Figure S3**. ^{13}C NMR spectrum of HECB in $CDCl_3$ 75 MHz.

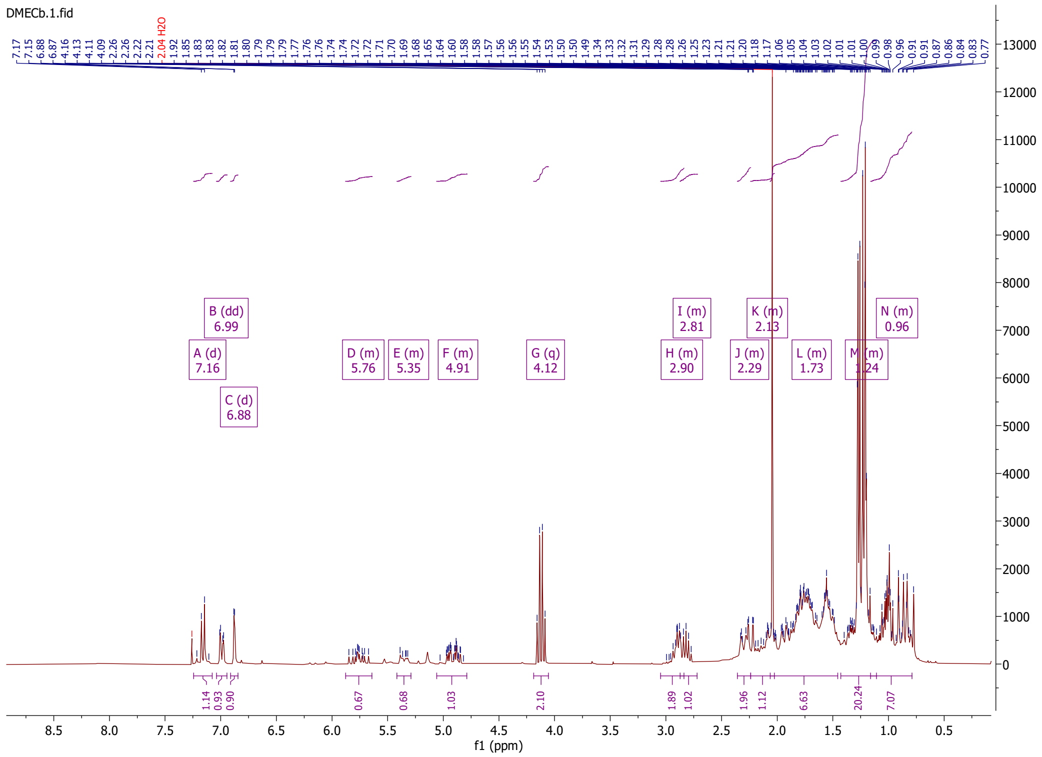

- **Figure S4**. ^{1}H NMR spectrum of DMECB in $CDCl_3$ 300 MHz.

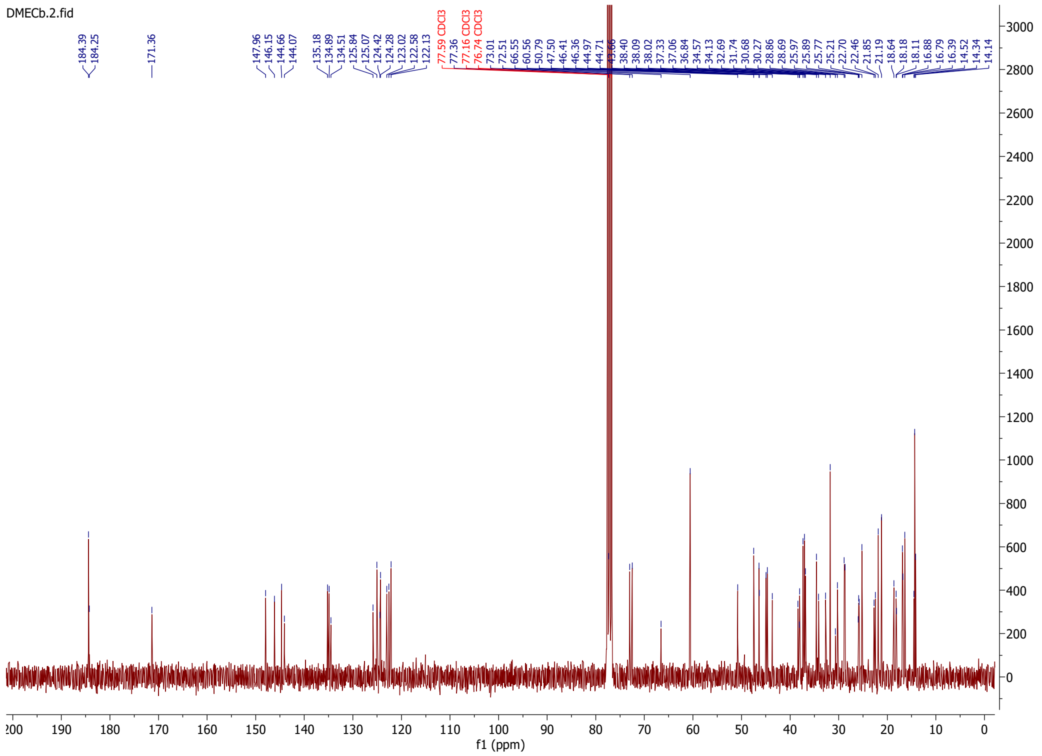

- **Figure S5**. ^{13}C NMR spectrum of DMECB in $CDCl_3$ 75 MHz.

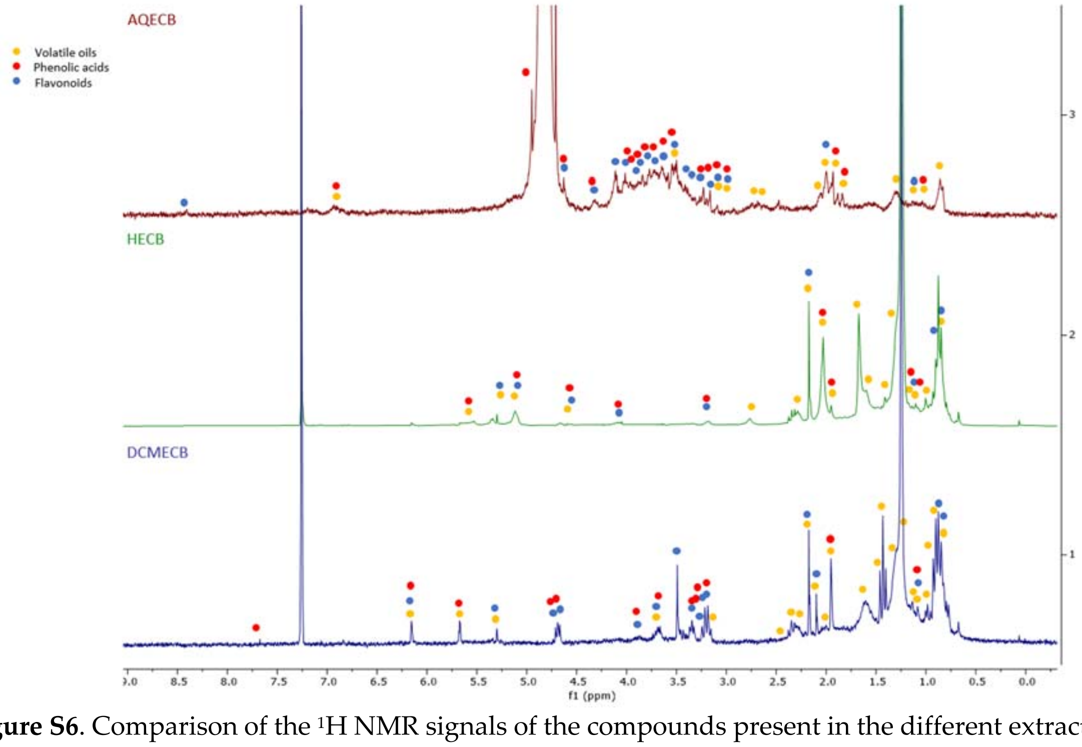

- **Figure S6**. Comparison of the ^{1}H NMR signals of the compounds present in the different extracts of *Clinopodium bolivianum* in the range of 0.0 to 9.0 ppm at 300 MHz. The symbols in the spectra correspond to the resonances of the main groups of marker compounds.

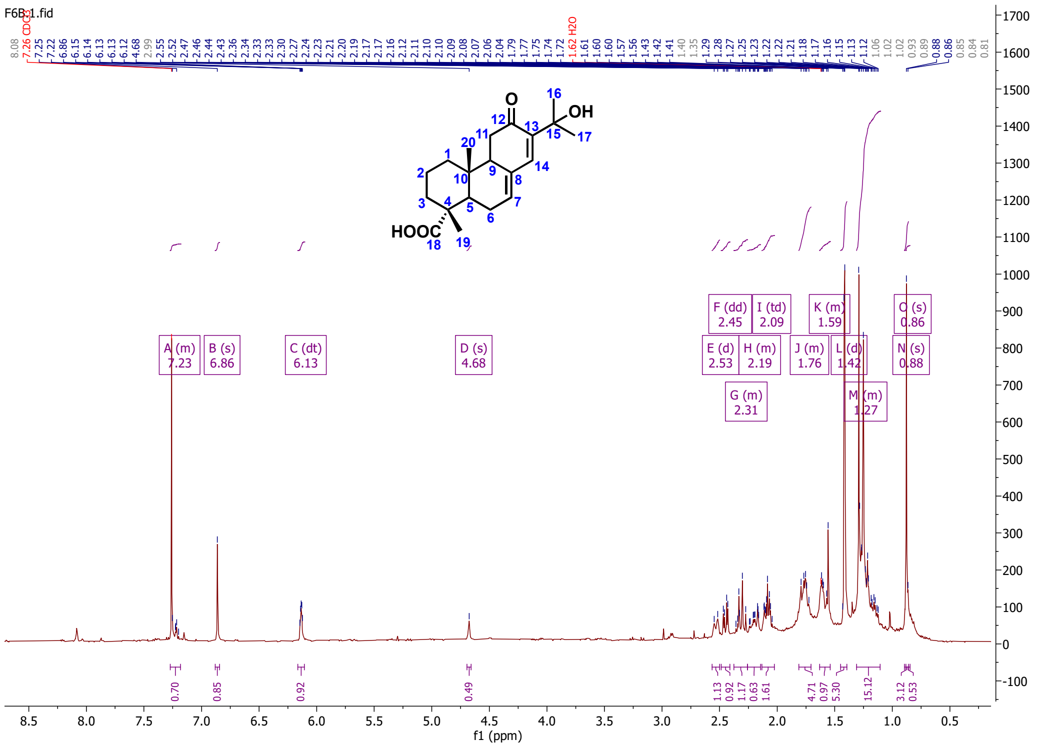

- **Figure S7**. ^{1}H NMR spectrum of 15-Hydroxy-12-oxo-abietic acid (**1**) in $CDCl_3$ 300 MHz.

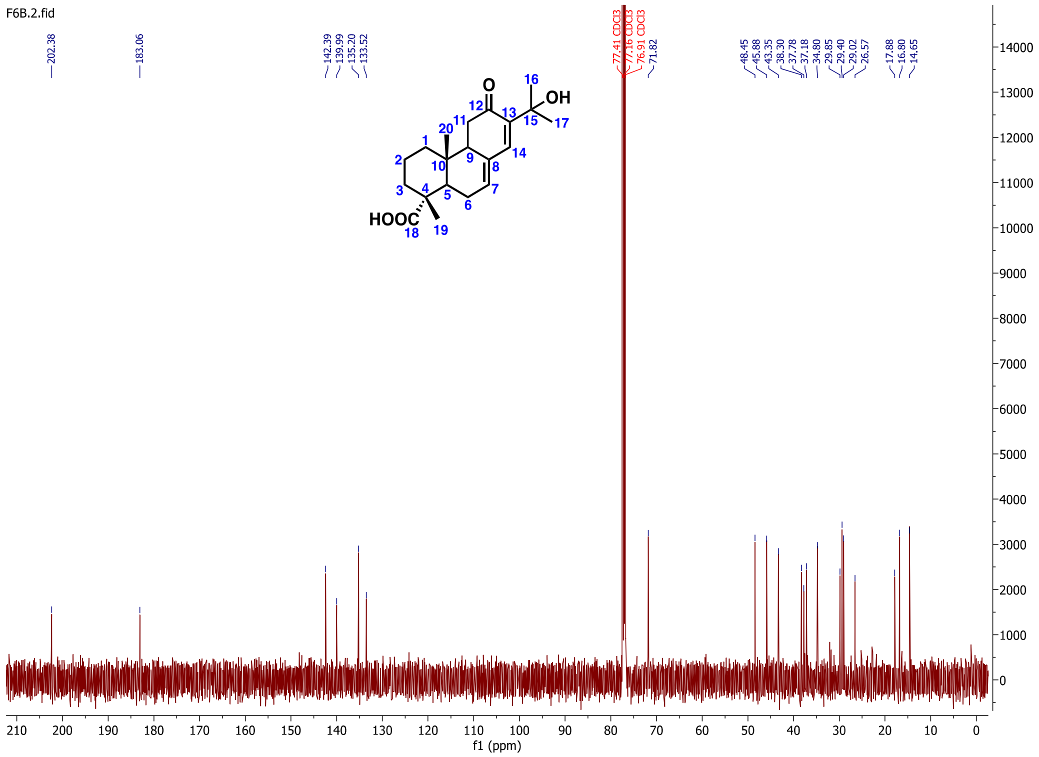

- **Figure S8**. ^{13}C NMR spectrum of 15-Hydroxy-12-oxo-abietic acid (**1**) in $CDCl_3$ 75 MHz.

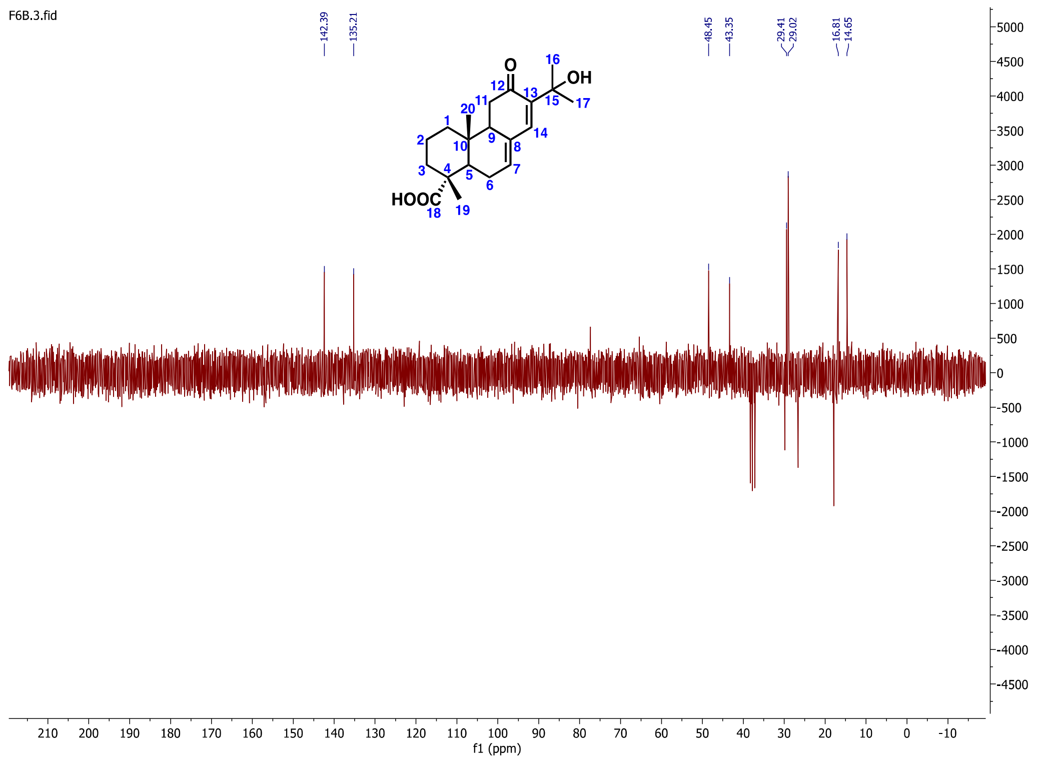

- **Figure S9**. DEPT-135 spectrum of 15-Hydroxy-12-oxo-abietic acid (**1**) in $CDCl_3$ 75 MHz.

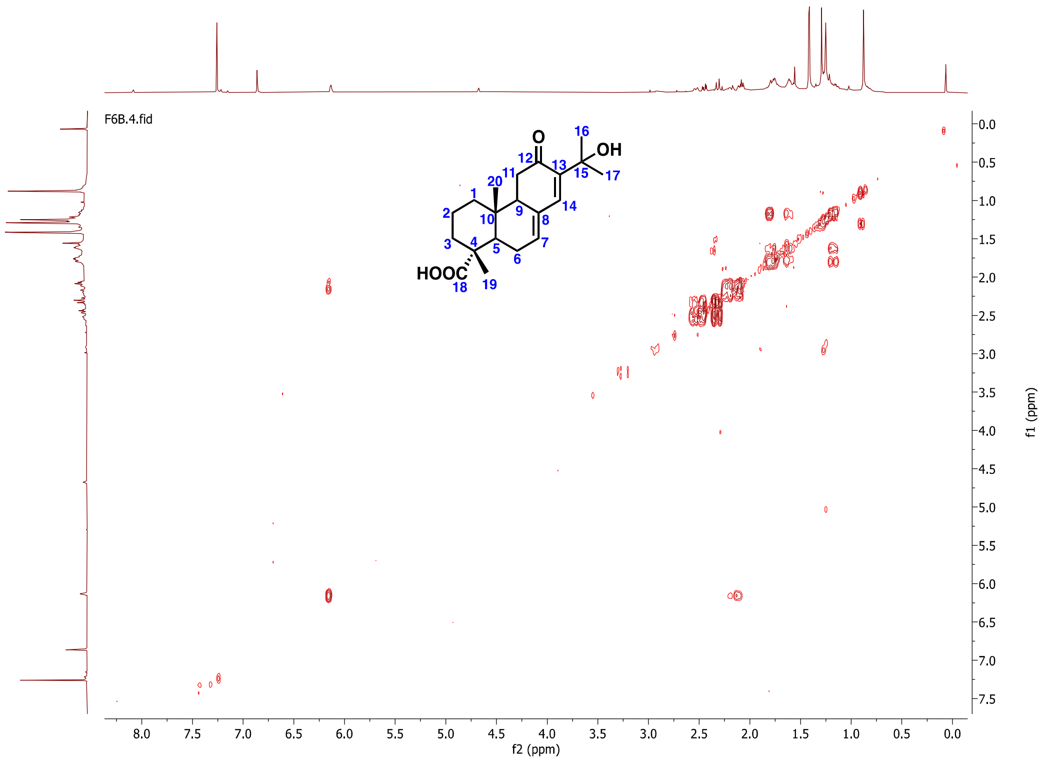

- **Figure S10**. ^1H-^1H COSY spectrum of 15-Hydroxy-12-oxo-abietic acid (**1**) in $CDCl_3$.

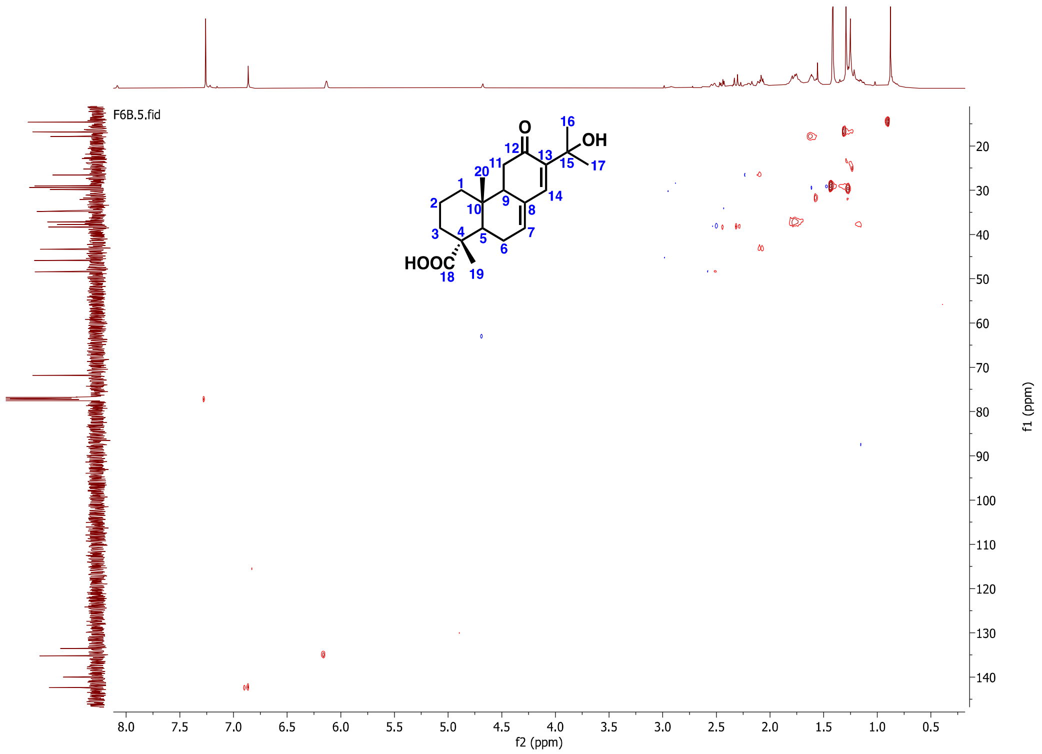

- **Figure S11**. ^{1}H-^{13}C HSQC spectrum of 15-Hydroxy-12-oxo-abietic acid (**1**) in $CDCl_3$.

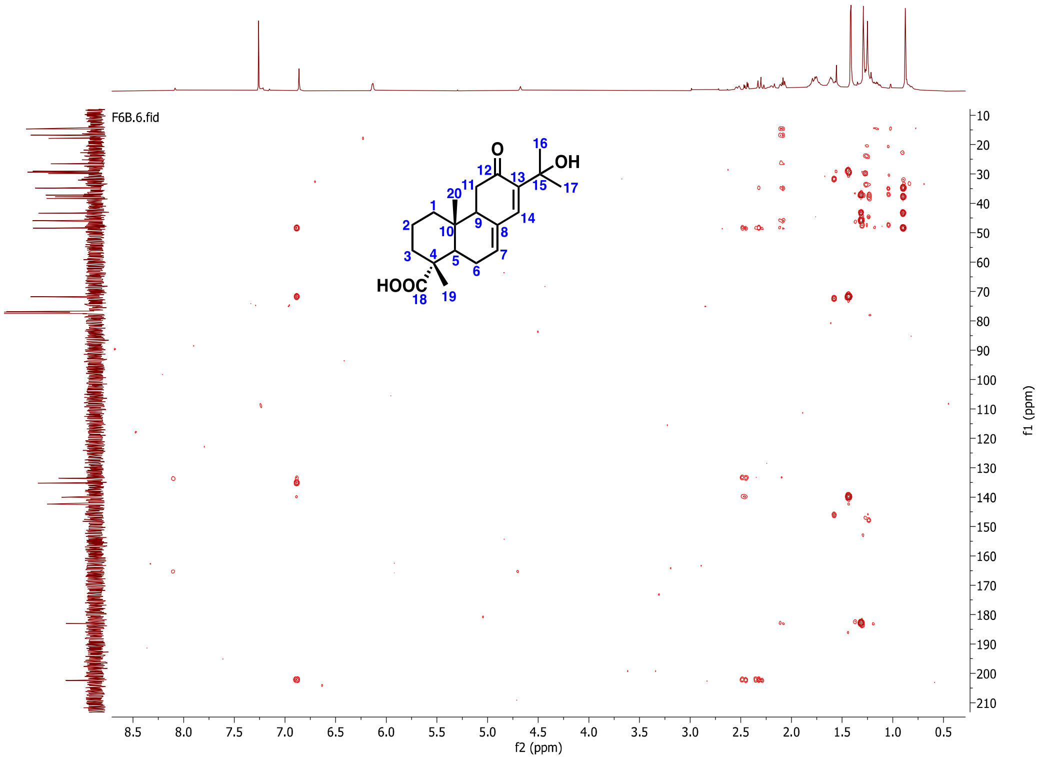

- **Figure S12**. ^{1}H-^{13}C HMBC spectrum of 15-Hydroxy-12-oxo-abietic acid (**1**) in $CDCl_3$.

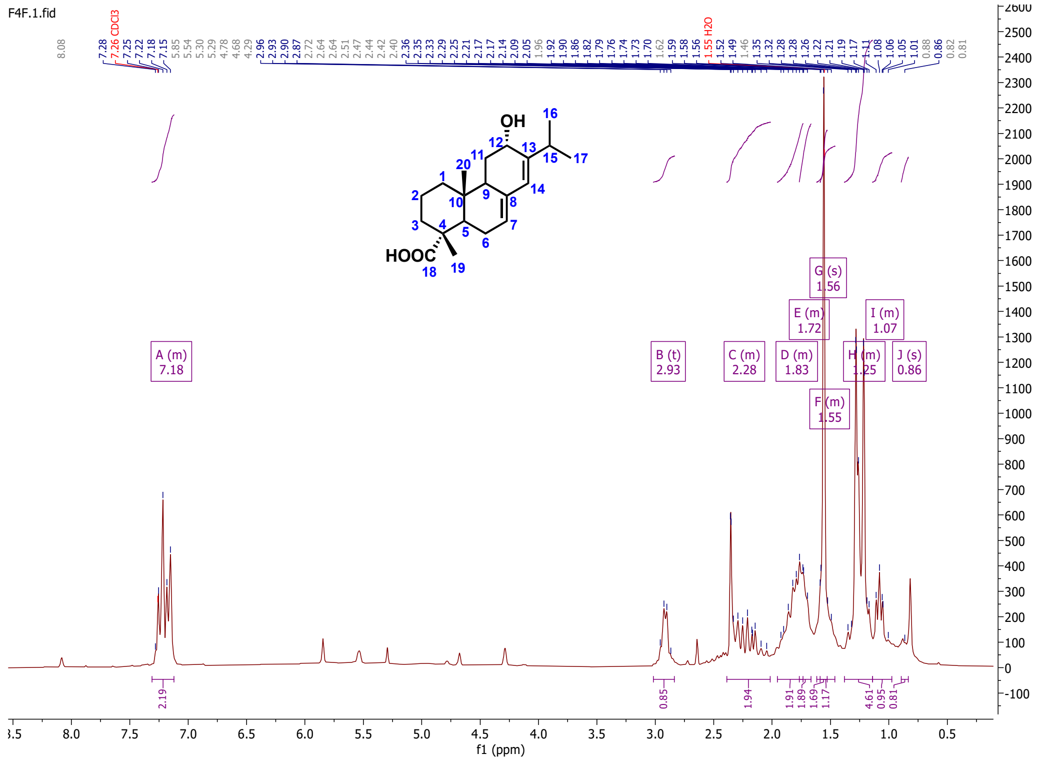

- **Figure S13**. ¹H NMR spectrum of 12α-Hydroxy-abietic acid (**2**) in $CDCl_3$ 300 MHz.

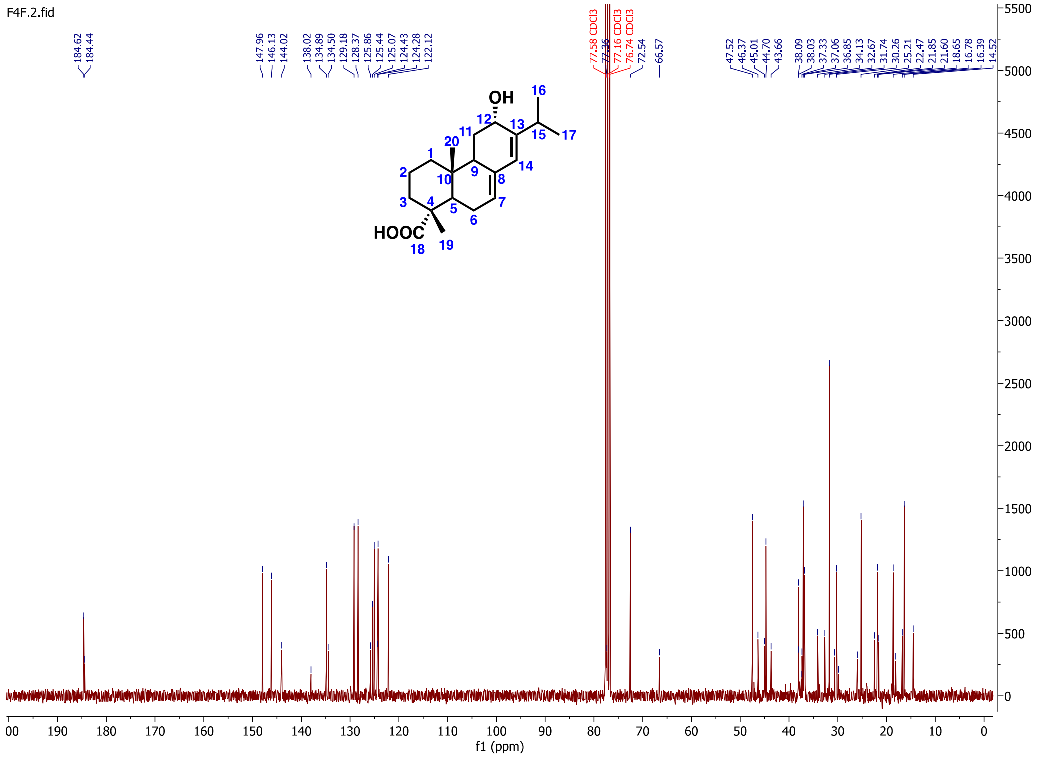

- **Figure S14**. ¹³C NMR spectrum of 12α-Hydroxy-abietic acid (**2**) in $CDCl_3$ 75 MHz.

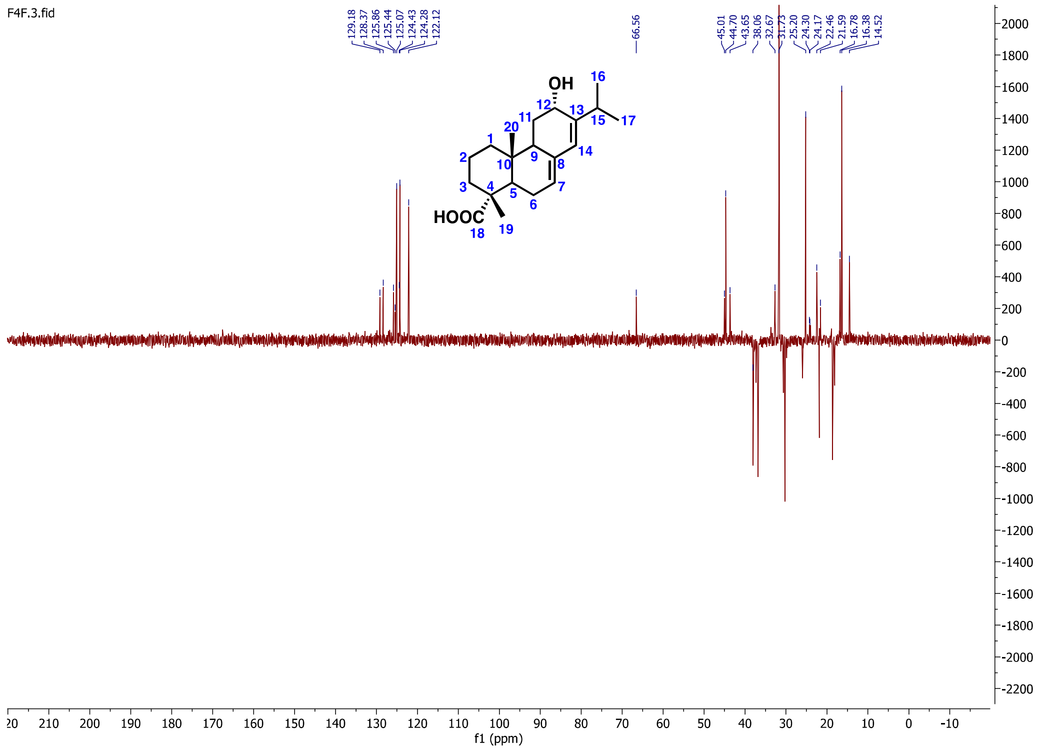

- **Figure S15**. DEPT-135 spectrum of 12α-Hydroxy-abietic acid (**2**) in CDCl3 75 MHz.

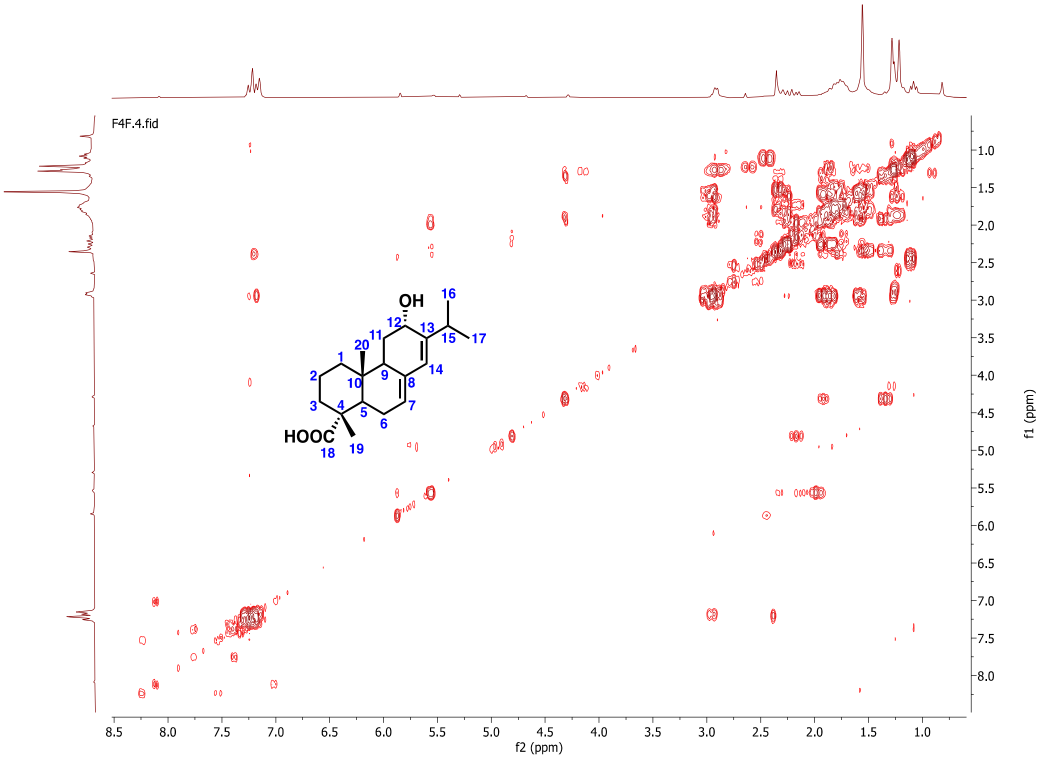

- **Figure S16**. ^{1}H-^{1}H COSY spectrum of 12α-Hydroxy-abietic acid (**2**) in CDCl3.

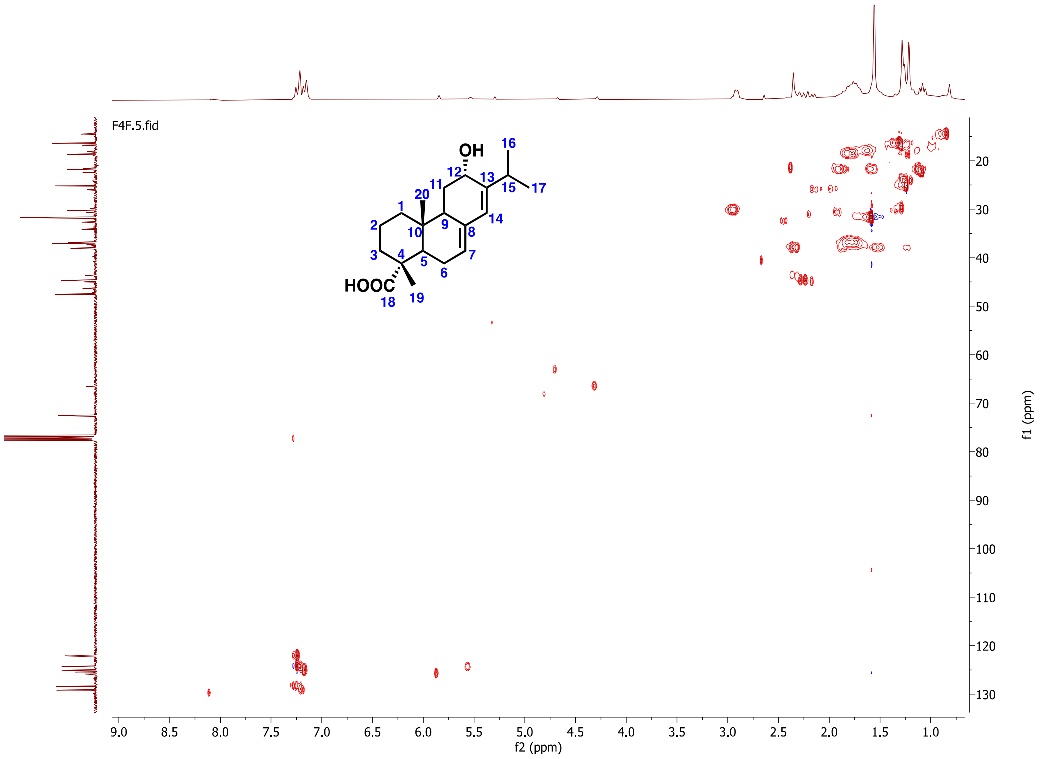

- **Figure S17**. ^{1}H-^{13}C HSQC spectrum of 12α-Hydroxy-abietic acid (**2**) in $CDCl_3$.

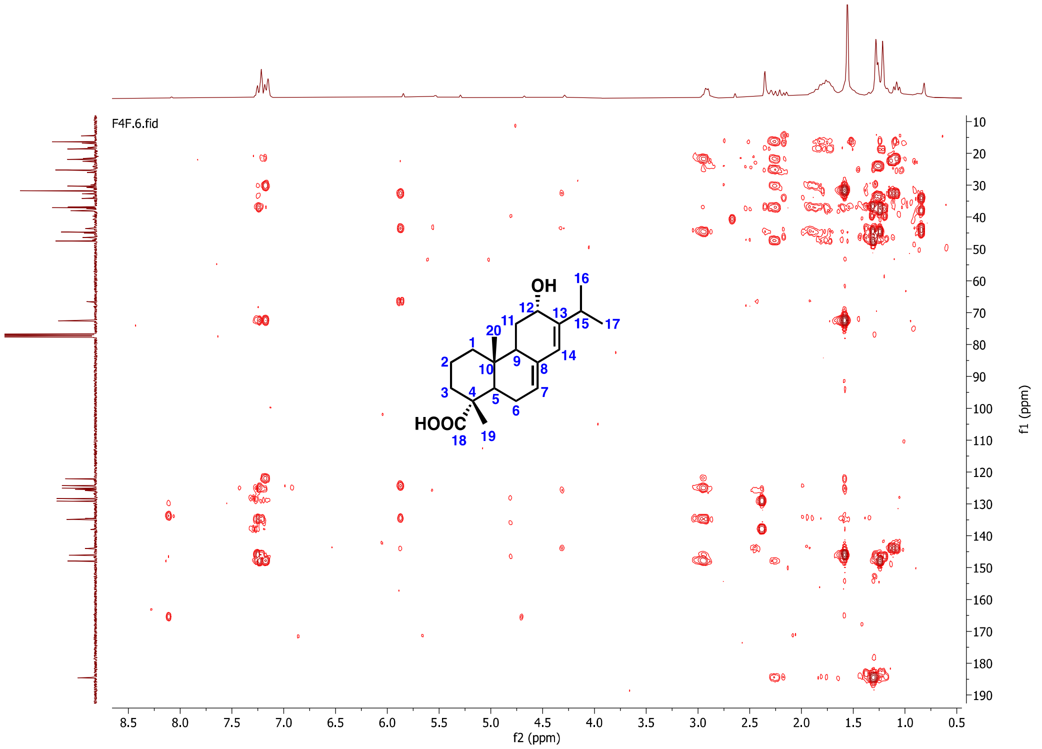

- **Figure S18**. ^{1}H-^{13}C HMBC spectrum of 12α-Hydroxy-abietic acid (**2**) in $CDCl_3$.

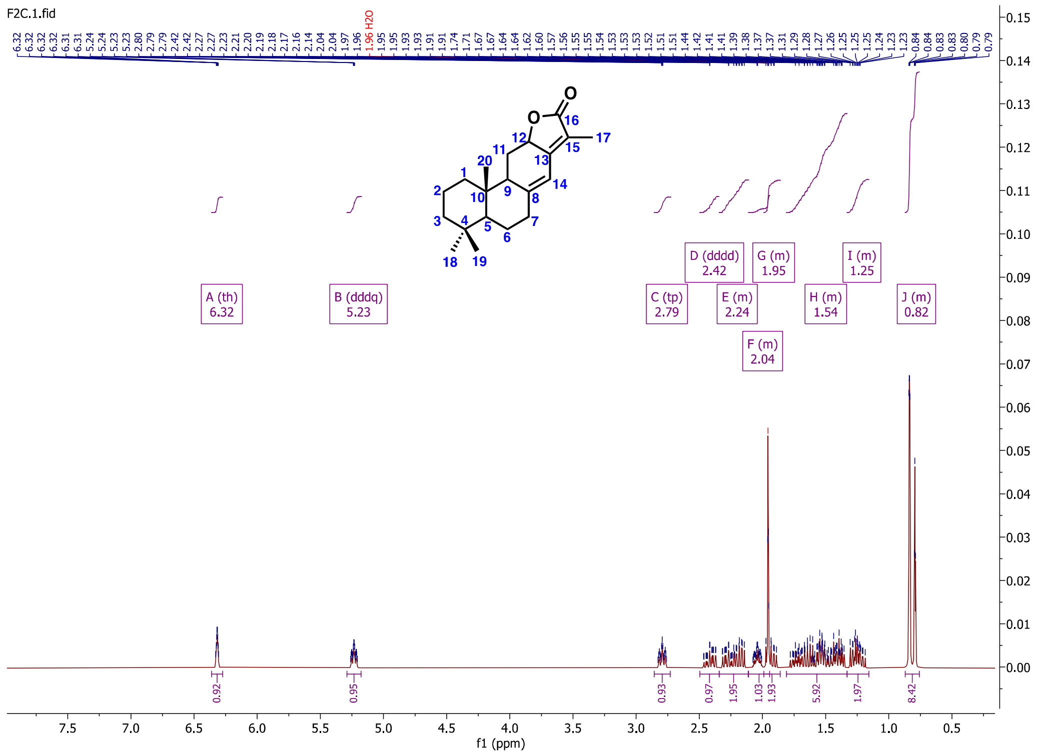

- **Figure S19**. ^{1}H NMR spectrum of (-)-Jolkinolide E (**3**) in $CDCl_3$ 300 MHz.

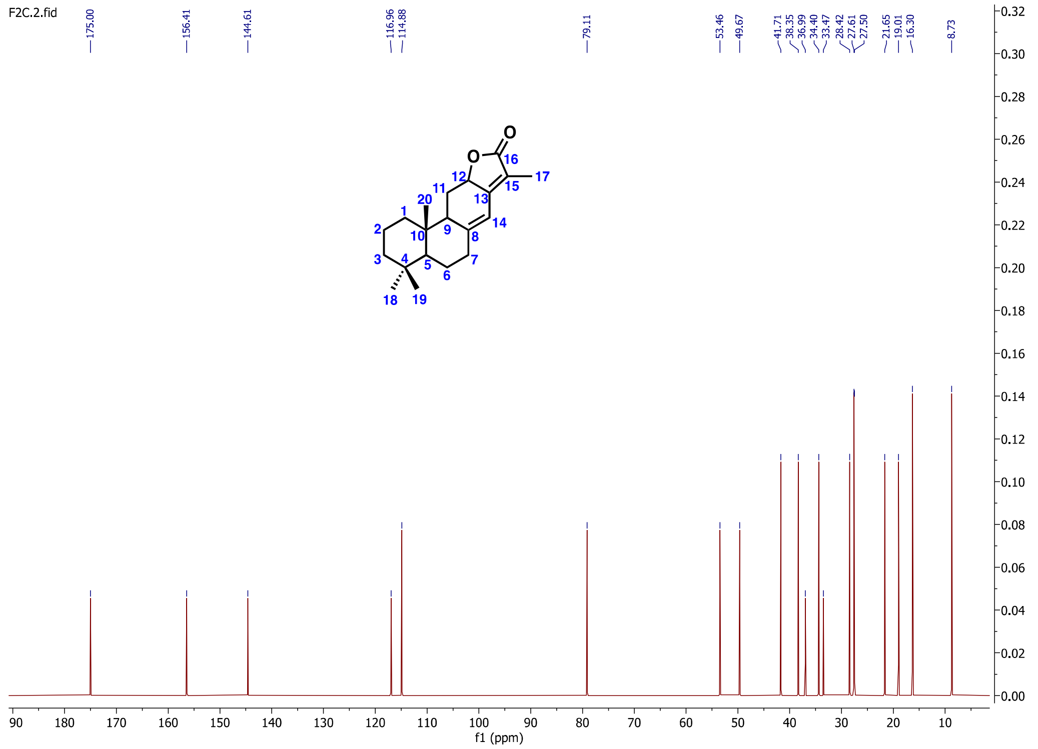

- **Figure S20**. ^{13}C NMR spectrum of (-)-Jolkinolide E (**3**) in $CDCl_3$ 75 MHz.

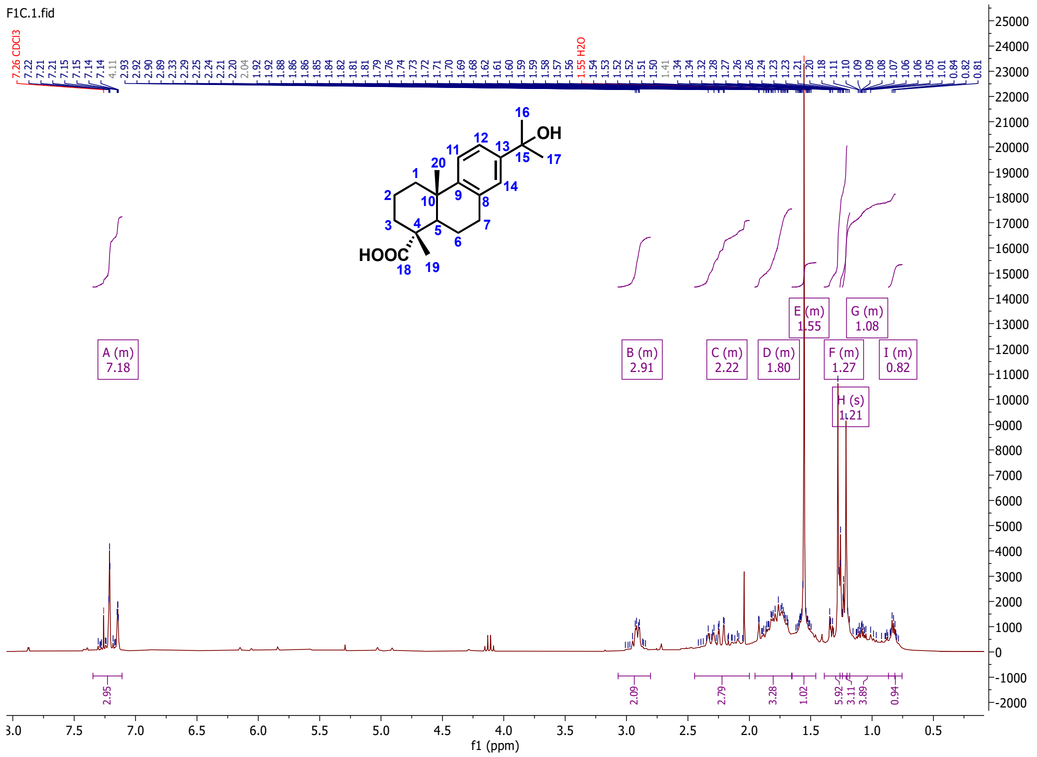

- **Figure S21**. ¹H NMR spectrum of 15-Hydroxy-dehydroabietic acid (**4**) in $CDCl_3$ 300 MHz.

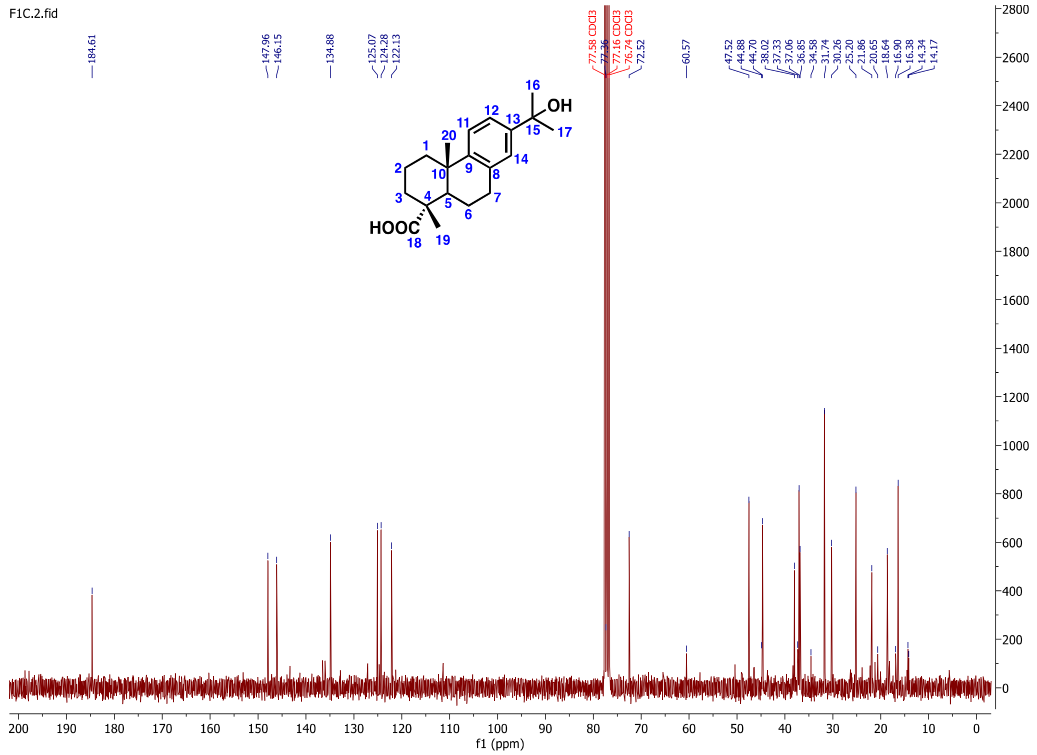

- **Figure S22**. ¹³C NMR spectrum of 15-Hydroxy-dehydroabietic acid (**4**) in $CDCl_3$ 75 MHz.

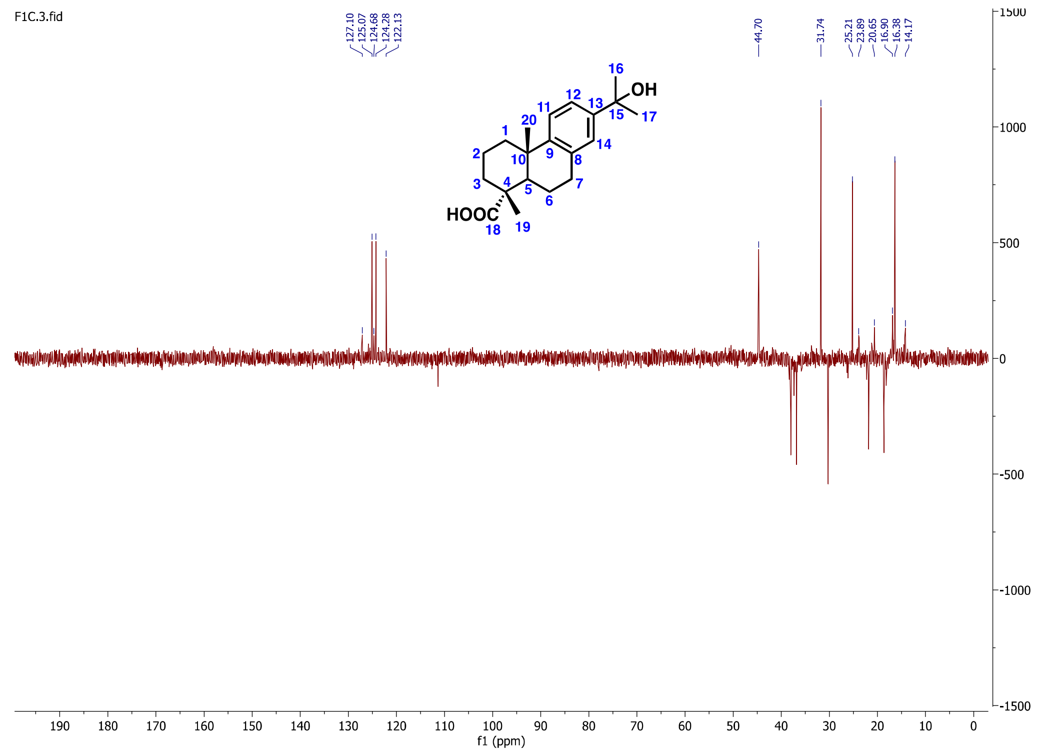

- **Figure S23**. DEPT-135 spectrum of 15-Hydroxy-dehydroabietic acid (**4**) in $CDCl_3$ 75 MHz.

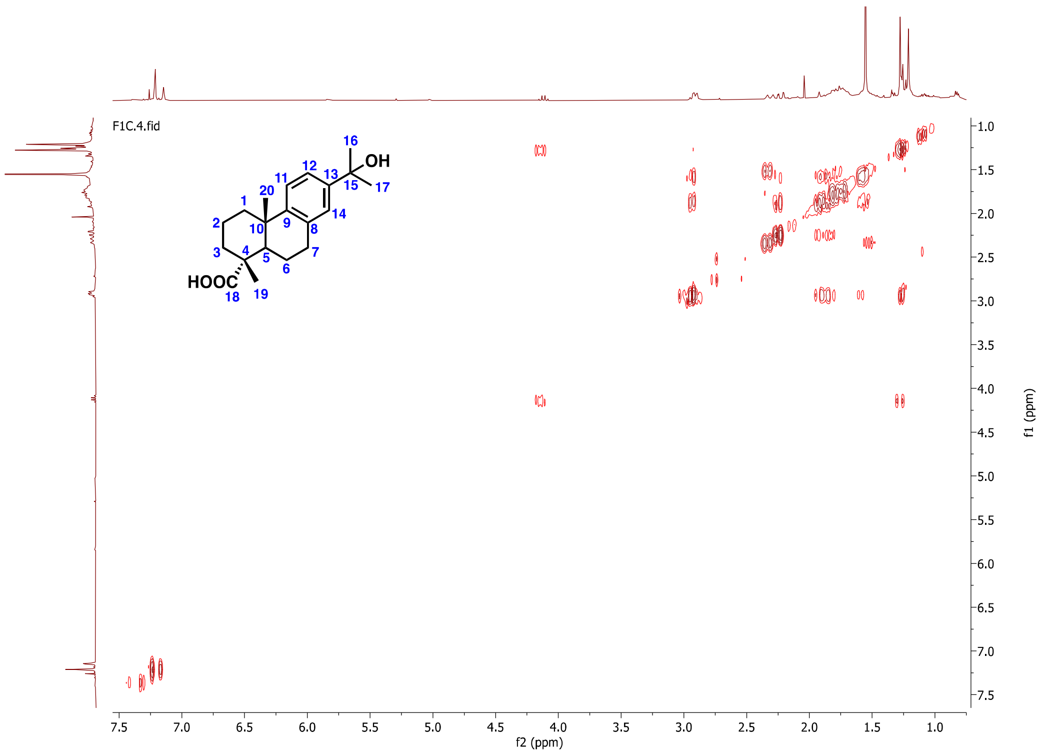

- **Figure S24**. ^1H-^1H COSY spectrum of 15-Hydroxy-dehydroabietic acid (**4**) in $CDCl_3$.

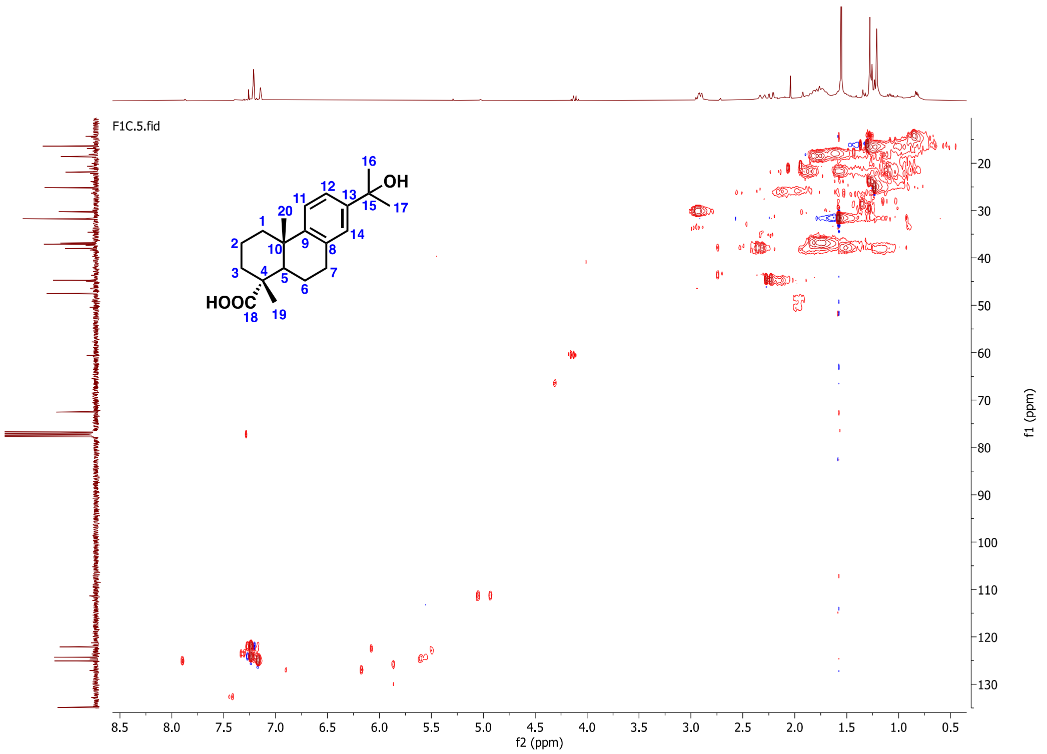

- **Figure S25**. ^{1}H-^{13}C HSQC spectrum of 15-Hydroxy-dehydroabietic acid (**4**) in $CDCl_3$.

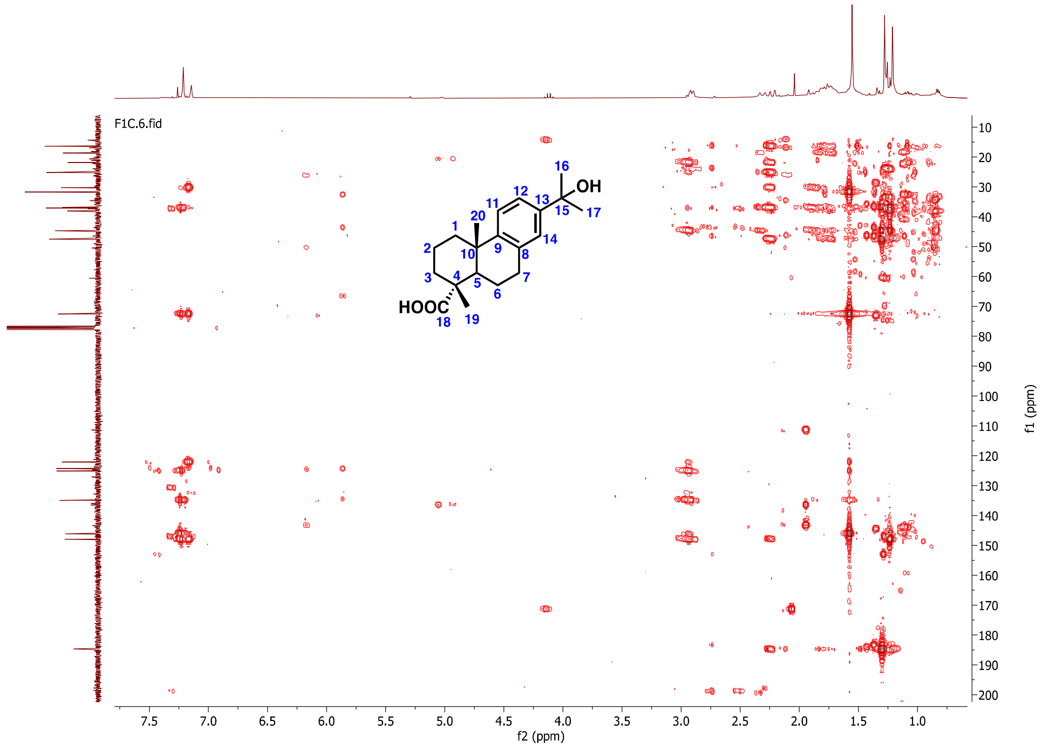

- **Figure S26**. ^{1}H-^{13}C HMBC spectrum of 15-Hydroxy-dehydroabietic acid (**4**) in $CDCl_3$.

- **Table S1**. Marker compounds of *C. bolivianum* identified in the ^{1}H NMR spectra of the different extracts.

**Terpenes**	**Polyphenols**	
**Volatile oils**	**Phenolic acids**	**Flavonoids**
Bicyclogermacrene: ^{1}H NMR (250 MHz, $CDCl_3$) δ_H 5.28-5.04 (m, H), 2.25-1.99 (m, 2H), 1.69-1.50 (m, 2H), 1.03 (t, *J*=1.5 Hz, 2H).	Caffeic acid-3-glucoside: ^{1}H NMR (250 MHz, $CDCl_3$) δ_H 9.94 (s, 1H), 7.81-7.73 (m, 1H), 7.65 (d, *J*=16.5 Hz, 1H), 7.08-6.94 (m, 2H), 6.70 (s, 1H), 6.34 (d, *J*=16.6 Hz, 1H), 4.95-4.81 (m, 1H), 4.80-4.66 (m, 1H), 4.70-4.57 (m, 1H), 4.56-4.43 (m, 1H), 3.98-3.83 (m, 1H), 3.78-3.62 (m, 2H), 3.59-3.36 (m, 3H*)*.	Apegenin-7-*O*-apioglucoside: ^{1}H NMR (250 MHz, $CDCl_3$) δ_H 12.66 (s, 0H), 8.57 (s, 0H), 7.92-7.80 (m, 1H), 7.01-6.89 (m, 1H), 6.73 (s, 0H), 6.51 (d, *J*=2.2 Hz, 0H), 6.38 (d, *J*=2.2 Hz, 0H), 5.25-5.09 (m, 1H), 4.79-4.65 (m, 1H), 4.25-3.70 (m, 6H), 3.62-3.36 (m, 1H).
α-Cadinol: ^{1}H NMR (250 MHz, $CDCl_3$) δ_H 2.30-1.85 (m, 1H), 1.85-1.62 (m, 1H), 1.69-1.52 (m, 1H), 1.58-1.18 (m, 1H), 1.30 (s, 1H), 0.83 (ddd, *J*=6.1, 4.7, 1.4 Hz, 2H).	Caffeic acid-3-rutinoside: ^{1}H NMR (250 MHz, $CDCl_3$) δ_H 9.94 (s, 1H), 7.81-7.73 (m, 1H), 7.65 (d, *J*=16.6 Hz, 1H), 7.08-6.94 (m, 2H), 6.70 (s, 1H), 6.34 (d, *J*=16.6 Hz, 1H), 6.17 (d, *J*=3.6 Hz, 1H), 5.71 (d, *J*=6.4 Hz, 1H), 5.15 (ddq, *J*=7.6, 3.6, 1.5 Hz, 1H), 4.89 (d, *J*=5.5 Hz, 1H), 4.68-4.45 (m, 4H), 4.19-4.06 (m, 2H), 3.93-3.73 (m, 2H), 3.70 (dt, *J*=8.3, 5.5 Hz, 1H), 3.59-3.44 (m, 2H), 3.44-3.19 (m, 2H), 1.96-1.74 (m, 1H), 1.05 (dt, *J*=6.7, 1.5 Hz, 3H).	Kaempferol-3-*O*-glycoside: ^{1}H NMR (250 MHz, $CDCl_3$) δ_H 12.51 (s, 1H), 10.90 (s, 1H), 8.96 (s, 1H), 7.89-7.77 (m, 1H), 6.97-6.85 (m, 1H), 6.32 (d, *J*=1.9 Hz, 1H), 6.21 (d, *J*=1.9 Hz, 1H), 5.45-5.32 (m, 1H), 4.65 (d, *J*=4.9 Hz, 1H), 4.09-3.91 (m, 2H), 3.97-3.84 (m, 1H), 3.79-3.63 (m, 1H), 3.70-3.50 (m, 1H).
trans-Caryophyllene: See Table S1.		Kaempferol-7-*O*-ramnoside: ^{1}H NMR (250 MHz, $CDCl_3$) δ_H 12.17 (s, 1H), 8.57 (s, 1H), 7.92-7.80 (m, 2H), 7.01-6.89 (m, 2H), 6.79-6.69 (m, 2H), 6.40 (d, *J*=2.1 Hz, 1H), 5.52 (d, *J*=6.1 Hz, 1H), 4.92 (d, *J*=5.3 Hz, 1H), 4.74 (d, *J*=4.8 Hz, 1H), 4.50 (d, *J*=5.3 Hz, 1H), 3.82 (ddd, *J*=7.3, 6.1, 5.2 Hz, 1H), 3.65 (td, *J*=7.3, 4.8 Hz, 1H), 3.54-3.39 (m, 1H), 3.39-3.22 (m, 1H), 1.13 (dd, *J*=5.0, 1.5 Hz, 2H).
1,8-Cineole: ^{1}H NMR (250 MHz, $CDCl_3$) δ_H 1.99-1.69 (m, 1H), 1.74-1.55 (m, 1H), 1.59-1.32 (m, 1H), 1.25 (d, *J*=1.5 Hz, 2H), 1.06 (s, 1H).		Eriodyctiol-7-*O*-glucoside: ^{1}H NMR (250 MHz, $CDCl_3$) δ_H 12.01 (s, 1H), 8.22 (s, 1H), 7.01 (d, *J*=1.8 Hz, 2H), 6.93-6.77 (m, 2H), 6.16-6.07 (m, 2H), 5.39 (ddt, *J*=7.0, 4.3, 0.7 Hz, 1H), 5.14-4.99 (m, 1H), 4.79-4.66 (m, 1H), 4.70-4.57 (m, 1H), 4.42-4.28 (m, 1H), 3.98-3.83 (m, 1H), 3.78-3.62 (m, 2H), 3.58-3.36 (m, 4H), 3.15 (dd, *J*=16.9, 4.3 Hz, 1H), 2.77 (dd, *J*=16.8, 6.9 Hz, 1H).
ρ-Cymene: ^{1}H NMR (250 MHz, $CDCl_3$) δ_H 7.18-6.99 (m, 4H), 2.86 (dpd, *J*=7.3, 6.6, 5.9 Hz, 1H), 1.27 (d, *J*=6.6 Hz, 6H).		Eriodyctiol-7-*O*-rutinoside: ^{1}H NMR (250 MHz, $CDCl_3$) δ_H 12.02 (s, 1H), 8.22 (s, 1H), 7.01 (d, *J*=1.8 Hz, 2H), 6.93-6.77 (m, 2H), 6.38 (d, *J*=2.2 Hz, 1H), 6.26 (d, *J*=2.2 Hz, 1H), 5.39 (ddt, *J*=7.0, 4.3, 0.7 Hz, 1H), 5.19-5.03 (m, 2H), 4.93-4.74 (m, 2H), 4.64-4.50 (m, 2H), 4.33 (td, *J*=4.6, 1.4 Hz, 3H), 4.08 (dd, *J*=11.0, 4.2 Hz, 1H), 3.92-3.73 (m, 3H), 3.69-3.46 (m, 5H), 3.15 (dd, *J*=16.9, 4.3 Hz, 1H), 2.77 (dd, *J*=16.8, 6.9 Hz, 1H), 1.26 (dd, *J*=4.9, 1.2 Hz, 3H).

Geranyl acetate: ^{1}H NMR (250 MHz, $CDCl_3$) δ_H 5.57 (h, *J*=1.4 Hz, 1H), 5.20-5.03 (m, 1H), 3.69 (s, 3H), 2.34-2.14 (m, 4H), 2.20-2.07 (m, 4H), 1.68-1.57 (m, 4H).

Germacrene D: ^{1}H NMR (250 MHz, $CDCl_3$) δ_H 5.20 (dddq, *J*=7.1, 4.4, 2.4, 1.3 Hz, 0H), 5.12-4.98 (m, 0H), 4.79-4.65 (m, 1H), 2.42-1.63 (m, 5H), 1.63-1.41 (m, 2H).

Linalool: ^{1}H NMR (250 MHz, $CDCl_3$) δ_H 5.90 (ddq, *J*=17.3, 11.6, 1.0 Hz, 1H), 5.23-5.05 (m, 2H), 2.14-1.93 (m, 1H), 1.69-1.61 (m, 2H), 1.68-1.59 (m, 2H), 1.65-1.54 (m, 1H), 1.59-1.40 (m, 1H), 1.29 (d, *J*=0.9 Hz, 2H).

Menthone: ^{1}H NMR (250 MHz, $CDCl_3$) δ_H 2.53 (dd, *J*=12.8, 6.9 Hz, 1H), 2.36-2.19 (m, 2H), 2.19-1.85 (m, 2H), 1.85-1.35 (m, 4H), 0.97 (d, *J*=6.5 Hz, 3H), 0.88 (ddd, *J*=10.6, 7.0, 1.5 Hz, 6H).

Pulegone: ^{1}H NMR (250 MHz, $CDCl_3$) δ_H 2.70-2.38 (m, 2H), 2.42-2.05 (m, 3H), 1.85 (dp, *J*=7.0, 1.2 Hz, 6H), 1.86-1.65 (m, 1H), 1.45 (dddd, *J*=12.7, 8.0, 6.2, 5.6 Hz, 1H), 0.98 (d, *J*=6.7 Hz, 3H).

Spathulenol: ^{1}H NMR (250 MHz, $CDCl_3$) δ_H 4.75 (dtt, *J*=8.9, 1.9, 1.0 Hz, 2H), 3.00 (s, 1H), 2.51-2.16 (m, 4H), 2.10-1.91 (m, 1H), 1.91-1.66 (m, 3H), 1.66-1.45 (m, 3H), 1.32 (d, *J*=1.5 Hz, 3H), 1.06 (dt, *J*=2.1, 1.5 Hz, 6H), 0.93 (dtp, *J*=7.8, 4.6, 1.5 Hz, 1H).

γ-Terpinene: ^{1}H NMR (250 MHz, $CDCl_3$) δ_H 5.56 (tq, *J*=4.3, 1.0 Hz, 1H), 5.38 (dddt, *J*=6.2, 4.3, 1.9, 1.0 Hz, 1H), 2.74-2.57 (m, 4H), 2.37 (ddtdd, *J*=12.8, 7.4, 6.4, 5.4, 1.1 Hz, 1H), 1.76-1.67 (m, 2H), 1.00 (d, *J*=6.4 Hz, 5H).

Thymol: ^{1}H NMR (250 MHz, $CDCl_3$) δ_H 7.16-7.05 (m, 1H), 6.75 (dtd, *J*=7.5, 1.4, 0.7 Hz, 1H), 6.58 (dd, *J*=2.1, 0.7 Hz, 1H), 5.24 (s, 1H), 3.18 (heptd, *J*=6.6, 0.6 Hz, 1H), 1.25 (d, *J*=6.6 Hz, 6H).

Luteolin-7-*O*-glycoside: ^{1}H NMR (250 MHz, $CDCl_3$) δ_H 12.17 (s, 1H), 8.09 (s, 1H), 7.48-7.36 (m, 2H), 6.96-6.84 (m, 2H), 6.75 (d, *J*=2.2 Hz, 1H), 6.48-6.36 (m, 2H), 5.13-5.00 (m, 1H), 4.77-4.66 (m, 1H), 4.70-4.57 (m, 1H), 4.42-4.28 (m, 1H), 3.98-3.83 (m, 1H), 3.78-3.62 (m, 2H), 3.58-3.36 (m, 4H).

Quercetin-3-*O*-soforoside: ^{1}H NMR (250 MHz, $CDCl_3$) δ_H 12.51 (s, 1H), 10.90 (s, 1H), 8.15 (s, 1H), 7.64-7.53 (m, 2H), 6.97 (s, 1H), 6.85-6.73 (m, 1H), 6.32 (d, *J*=1.9 Hz, 1H), 6.21 (d, *J*=1.9 Hz, 1H), 5.77-5.64 (m, 1H), 4.95-4.86 (m, 1H), 4.72-4.56 (m, 2H), 4.50 (dd, *J*=5.0, 0.9 Hz, 1H), 4.40 (d, *J*=6.3 Hz, 1H), 4.32-3.91 (m, 6H), 3.90-3.59 (m, 4H), 3.66-3.38 (m, 3H).
